# Longitudinal phenotypic aging metrics in the Baltimore Longitudinal Study of Aging

**DOI:** 10.1038/s43587-022-00243-7

**Published:** 2022-07-18

**Authors:** Pei-Lun Kuo, Jennifer A. Schrack, Morgan E. Levine, Michelle D. Shardell, Eleanor M. Simonsick, Chee W. Chia, Ann Zenobia Moore, Toshiko Tanaka, Yang An, Ajoy Karikkineth, Majd AlGhatrif, Palchamy Elango, Linda M. Zukley, Josephine M. Egan, Rafael de Cabo, Susan M. Resnick, Luigi Ferrucci

**Affiliations:** 1 Translational Gerontology Branch, National Institute on Aging, National Institutes of Health, Baltimore, MD, USA.; 2 Department of Epidemiology, Johns Hopkins Bloomberg School of Public Health, Baltimore, MD, USA.; 3 Department of Pathology, Yale University School of Medicine, New Haven, CT, USA.; 4 Department of Epidemiology and Public Health and Institute for Genome Sciences, University of Maryland School of Medicine, Baltimore, MD, USA.; 5 Laboratory of Clinical Investigation, National Institute on Aging, National Institutes of Health, Baltimore, MD, USA.; 6 Laboratory of Behavioral Neuroscience, National Institute on Aging, National Institutes of Health, Baltimore, MD, USA.; 7 Laboratory of Cardiovascular Science, National Institute on Aging, National Institutes of Health, Baltimore, MD, USA.; 8 Clinical Research Core, National Institute on Aging, National Institutes of Health, Baltimore, MD, USA.

## Abstract

To define metrics of phenotypic aging, it is essential to identify biological and environmental factors that influence the pace of aging. Previous attempts to develop aging metrics were hampered by cross-sectional designs and/or focused on younger populations. In the Baltimore Longitudinal Study of Aging (BLSA), we collected longitudinally across the adult age range a comprehensive list of phenotypes within four domains (body composition, energetics, homeostatic mechanisms and neurodegeneration/neuroplasticity) and functional outcomes. We integrated individual deviations from population trajectories into a global longitudinal phenotypic metric of aging and demonstrate that accelerated longitudinal phenotypic aging is associated with faster physical and cognitive decline, faster accumulation of multimorbidity and shorter survival. These associations are more robust compared with the use of phenotypic and epigenetic measurements at a single time point. Estimation of these metrics required repeated measures of multiple phenotypes over time but may uniquely facilitate the identification of mechanisms driving phenotypic aging and subsequent age-related functional decline.

Aggressive control of risk factors, improved standard of living and progress in the quality and delivery of health care have all contributed to improved health and greater life expectancy in the world’s population^1^. These advances, however, are counterbalanced by trends toward a disproportionate expansion of the period of life characterized by diseases and disability in relationship to total life expectancy^2,3^. Disease-specific prevention and early diagnosis strategies, cornerstones of modern medicine, contribute to longer life expectancy but only marginally extend health span^2,3^. A complementary approach to improving health in the rapidly aging population is to recognize that increasing age is the single most important risk factor for most chronic diseases and adverse health outcomes^4,5^. Indeed, there is substantial heterogeneity in the accumulation of health and functional problems over the lifespan, and such heterogeneity is due to environmental and genetic differences that modulate the rate of aging. Studies in model organisms demonstrate that the rate of biological aging can be tweaked not only by genetic manipulation but also by behavioral and pharmacological interventions^5-9^. To this end, a robust measure that defines the rate of phenotypic aging independent of chronological age would be an extremely useful benchmark to identify intrinsic mechanisms of biological aging. In addition, such a measure could help to identify ‘accelerated agers’ and target them for personalized preventive strategies aimed at slowing down and/or modifying the consequences of accelerated aging.

Previous attempts to quantify phenotypic aging using the weighted average of laboratory values or clinical phenotypes have been successful in creating global scores that, independent of chronological age, predict health outcomes ^9-16^. However, with few exceptions, previous work was (1) based on cross-sectional data, which can be biased by informative censoring and cannot account for variation in individual set-points within the normal or near-normal range (for example, vegetarians have low normal hemoglobin levels that are not pathologic) or (2) included only individuals within a narrow age range, which may limit generalizability to older adults^12-14,16^. Other composite scores, such as frailty measures, rely on disease burden, impairments and functional limitations^17,18^. Although these are powerful biomarkers of health and prognosis in older persons, such composite scores are much less informative in younger individuals who have no clinical evidence of disease due to effective resilience mechanisms that mask the existence and effects of pathology^4,19,20^. Indeed, we have previously argued that substantial delay exists between damage accumulation at the biological level and the emergence of phenotypic and functional manifestations of aging^4,6,21^. In the work that follows, we aim to demonstrate that analysis of longitudinal change in phenotypic traits enables the capture of subtle differences in phenotypic aging at the time when young, middle-aged and even older adults are relatively free of disease and impairment and there is still great potential for personalized preventive intervention.

Using data from the BLSA, a continuous enrollment cohort study of healthy aging across the life course, we previously demonstrated that age-related rates of change in phenotypical manifestations of aging within and across four phenotypic domains—body composition, energetics, homeostatic mechanism and neurodegeneration/neuroplasticity—show characteristic and heterogeneous linear and nonlinear longitudinal trajectories over the adult lifespan^6^. Recently, we proposed a hierarchical framework that incorporates biological, phenotypic and functional metrics of aging to advance geroscience research (Fig. 1)^6,21^. In the proposed framework, the phenotypic manifestations of aging stem from the mechanisms of aging biology and cause deterioration in both health and cognitive and physical function that occur in most aging individuals, although with heterogeneous schedules. Based on the proposed framework, the project presented aimed to: (1) develop a global longitudinal phenotypic score and (2) evaluate its association with changes in health and functional outcomes.

We started the development of a longitudinal phenotypic score of aging by combining information on individual deviations from linear and nonlinear longitudinal population trajectories of different phenotypes (Figs. 2 and 3). Then, using data from 968 BLSA participants ranging in age from their 20s to 90s at baseline, and with a total of 4,851 follow-up visits, we evaluated whether, independent of confounders, this global longitudinal phenotypic score was associated with longitudinal rates of change in physical and cognitive function, accumulation of multimorbidity and lifespan. Our hypothesis is that individuals who show decelerated rates of phenotypic change experience slower physical and cognitive decline over follow-up, less accumulation of multimorbidity and lower mortality. This approach is consistent with our a priori hypothesis of a hierarchical relationship between phenotypic and functional aspects of aging^21^.

## Results

### Do aging rates differ across the adult age span?

**Fig. 2 ∣:**
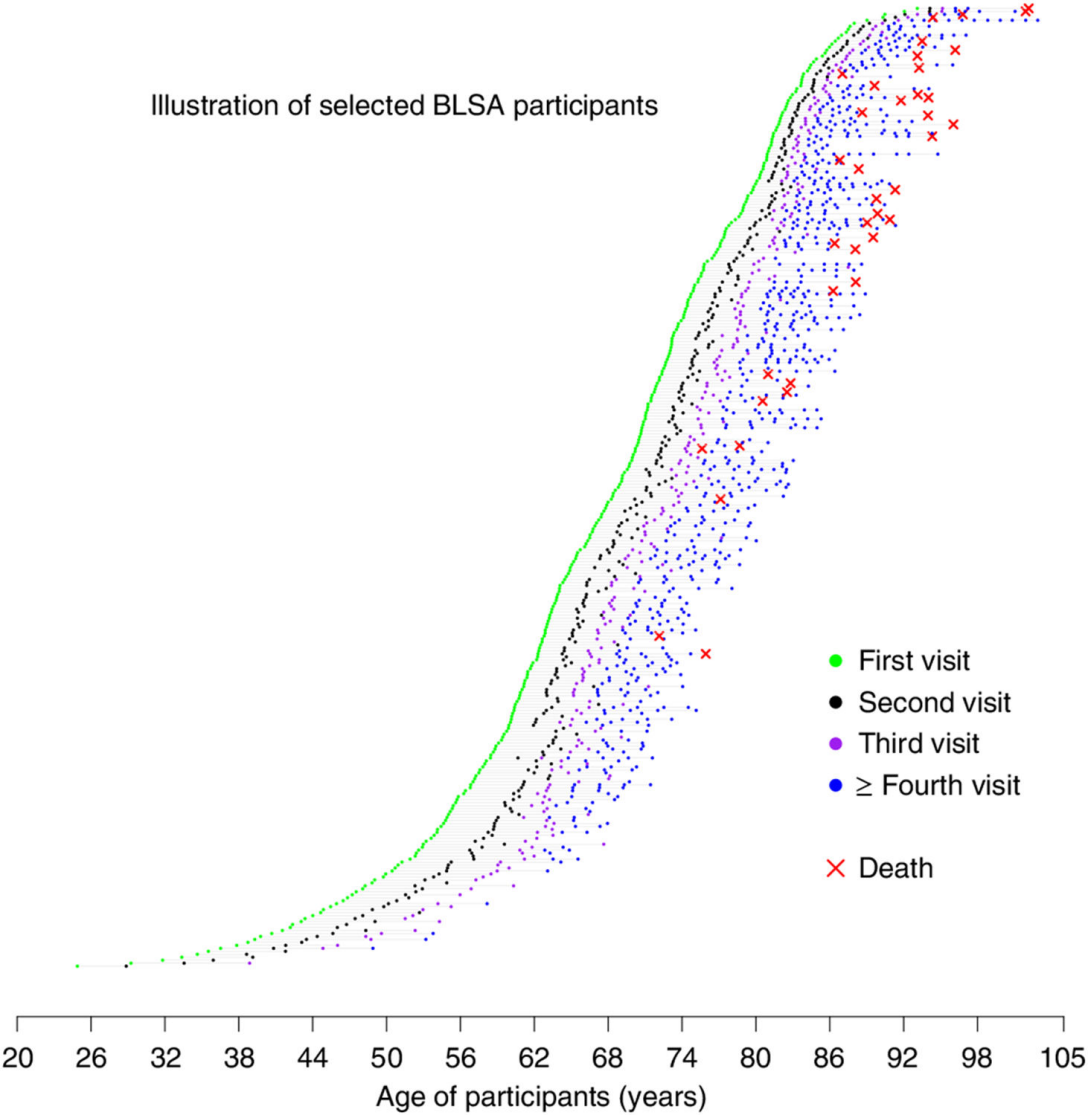
The BLSA analytic cohort. Random sample of the BLSA cohort used for our analyses. Participants had a wide age range at the time of enrollment, and follow-up duration. Scheduled follow-up intervals were age dependent, ranging from 4 years for those <60 years, 2 years for those aged 60-79 years to 1 year for those aged ≥80 years.

**Fig. 3 ∣:**
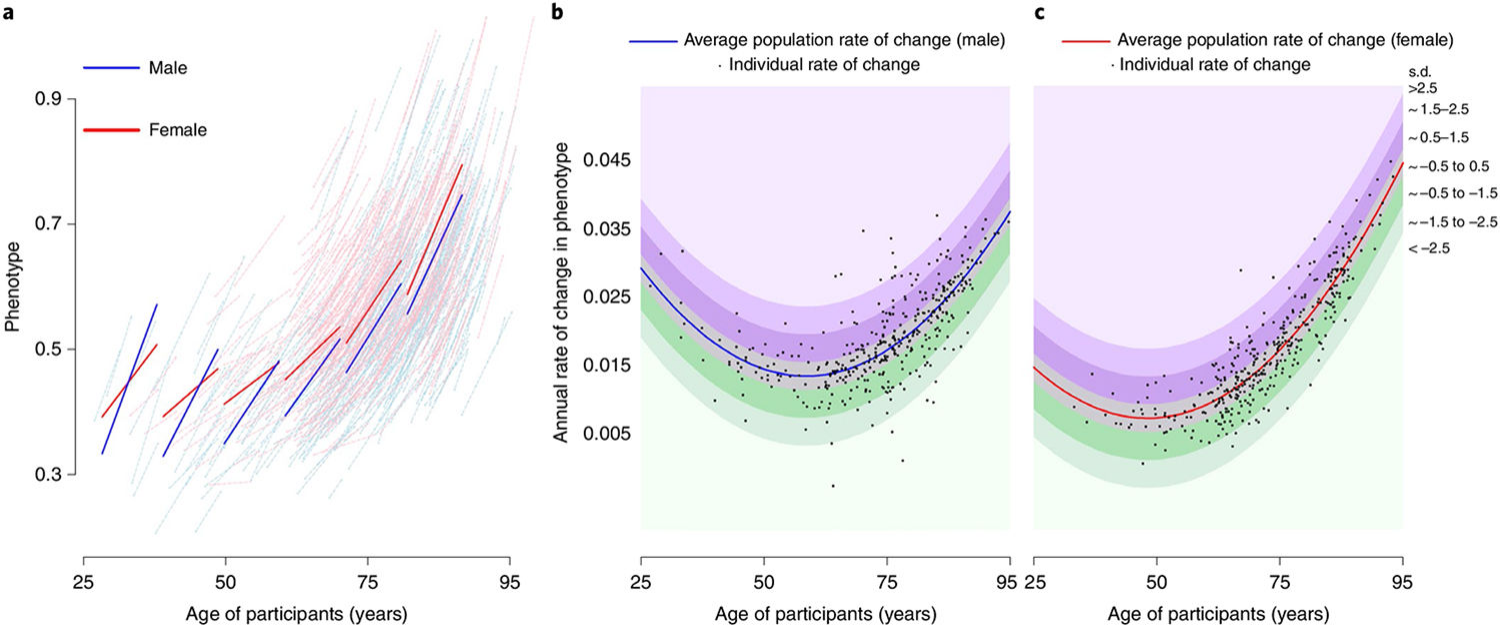
Relationship between average rate of change in the population of a specific phenotype and rate of change of the same phenotype in individual BLSA participants. **a–c**, The example reported in this figure uses the cost/capacity ratio, one of the phenotypes in the energetics domain, operationalized as the ratio between the energy cost of slow walking (ml kg^−1^ min^−1^) and energy capacity measured by peak oxygen consumption during a 400-m walk (ml kg^−1^ min^−1^) (detailed description in Supplementary Methods I). Number of participants, 755 (male 378, female 377). **a**, Spaghetti plot of longitudinal changes in cost-to-capacity ratio in men and women at the population level (thick blue and red lines, respectively) and for individual participants (thin lines), estimated from mixed-effect models (Methods and Supplementary Methods III). **b**,**c**, Estimated rates of change in cost/capacity ratio are depicted for individual participants (black dots; **b**, males; **c**, females) at their age of study entry. Bands of different color indicate how far the individual rates of change diverge from the population rate of change, expressed as s.d. Of note, because the rate of reference varies at different ages, a specific rate of change conveys information on accelerated or decelerated aging only when the specific age of the participant is considered.

Of the 968 participants included in this analysis, 512 (52.9%) were women and baseline age ranged between 24.9 and 93.7 years, with a median follow-up of around 7–9 years (Fig. 2, Supplementary Table 1 and Supplementary Fig. 1). We characterized sex-specific, population-based, longitudinal trajectories of several aging phenotypic traits grouped into four domains—body composition, energetics, homeostatic mechanisms and neurodegeneration/neuroplasticity. By fitting a family of polynomial regressions, we previously demonstrated that some of these age trajectories are linear (interleukin-6, c-reactive protein, albumin, red blood cell distribution width) while others are nonlinear (waist circumference, waist/height ratio, body mass index, lean mass, appendicular lean mass, fat mass, mid-thigh area, resting metabolic rate, peak oxygen consumption during treadmill testing, peak oxygen consumption during 400-m walk, cost/capacity ratio, forced expiratory volume in the first second (FEV1), forced vital capacity (FVC), FEV1/FVC, hemoglobin, absolute neutrophil count, fasting glucose, pulse pressure, systolic blood pressure, diastolic blood pressure, carotid/femoral pulse wave velocity, creatinine clearance, total cholesterol, low-density lipoproteins, high-density lipoproteins, triglyceride, total brain volume, white matter volume, gray matter volume, ventricular volume, nerve conduction velocity), and trajectories in men and women generally differ (Fig. 3 and Supplementary Figs. 2 and 3). Specifically, we calculated for each phenotype the difference between an individual’s rate of change and the sex-specific average rate of change in the population at the baseline age of that participant (Fig. 3). Of note, because some phenotypes show nonlinear trajectories over the lifespan, the population rate of change used for comparison may differ depending on the participant’s age.

Further, we standardized these values and averaged them within each domain. For each domain, higher scores indicate faster longitudinal age-phenotypic changes (that is, accelerated aging) while lower scores represent slower longitudinal age-phenotypic changes compared with the overall population at the same age of the participant. Longitudinal scores in four domain-specific phenotypes—body composition, energetics, homeostatic mechanisms and neuroplasticity/neurodegeneration—were symmetrically distributed across the age range (Supplementary Fig. 4). These findings suggest that, even in a cohort of healthy individuals, there exists wide heterogeneity of phenotypic changes from adulthood to late life. Correlations between the four domain-specific longitudinal phenotypic scores are modest (∣correlation∣ ranging between 0.03 and 0.10), suggesting that substantial intra-individual heterogeneity in the rate of aging exists across phenotypic domains and justifying the need to combine information across domains. Finally, the standardized scores were averaged into a global, longitudinal phenotypic score that represents the rate of phenotypic aging compared with that of the general population.

Several lines of research indicate that health and quality of life in older persons are best assessed through measures of physical and cognitive function, in addition to disease diagnoses and clinical signs or symptoms^22,23^. Both physical and cognitive function strongly predict ‘hard’ health outcomes including loss of autonomy and death^22,24^. Based on these considerations, we validated our global longitudinal phenotypic score by evaluating its correlations with rates of change in physical and cognitive functions.

### Longitudinal phenotypic aging and change in physical function.

**Fig. 4 ∣:**
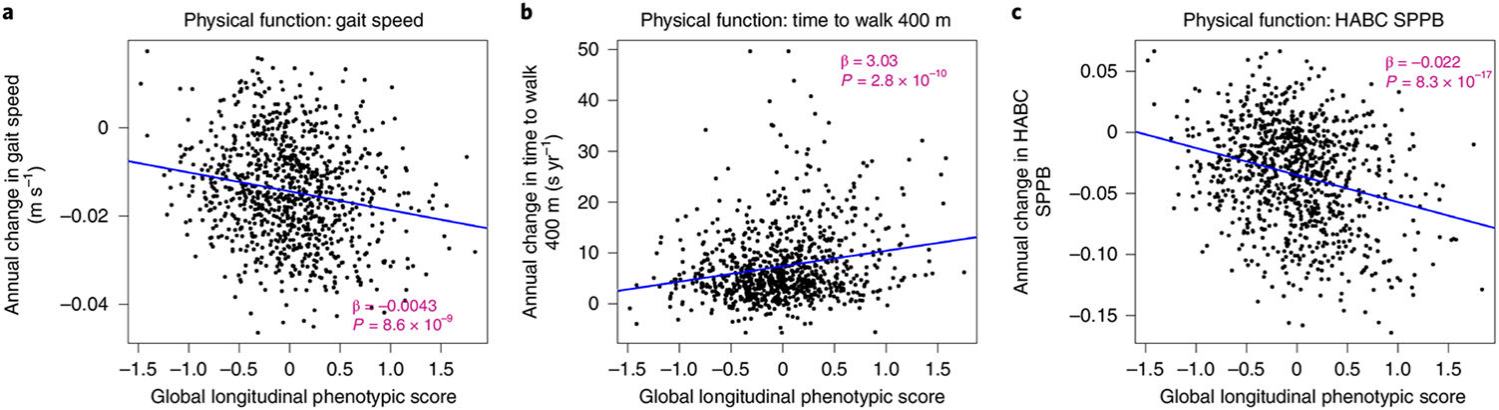
Relationship between global longitudinal phenotypic score and change in physical functions for three mobility tests. **a**–**c**, Higher global longitudinal phenotypic scores indicate accelerated phenotypic aging trajectories. Higher annual decrease in gait speed (**a**) and HABC SPPB scores (**c**), along with higher annual increase in the time to walk 400 m (**b**), indicate faster decline of physical function. Two-sided tests were used, and the displayed *P* values were not adjusted for multiple comparisons. Participants with at least two visits, *n* = 951; usual gait speed, *n* = 904; time to finish 400-m walk, *n* = 950 (HABC SPPB); also see Supplementary Table 1b for more details.Source data

**Fig. 5 ∣:**
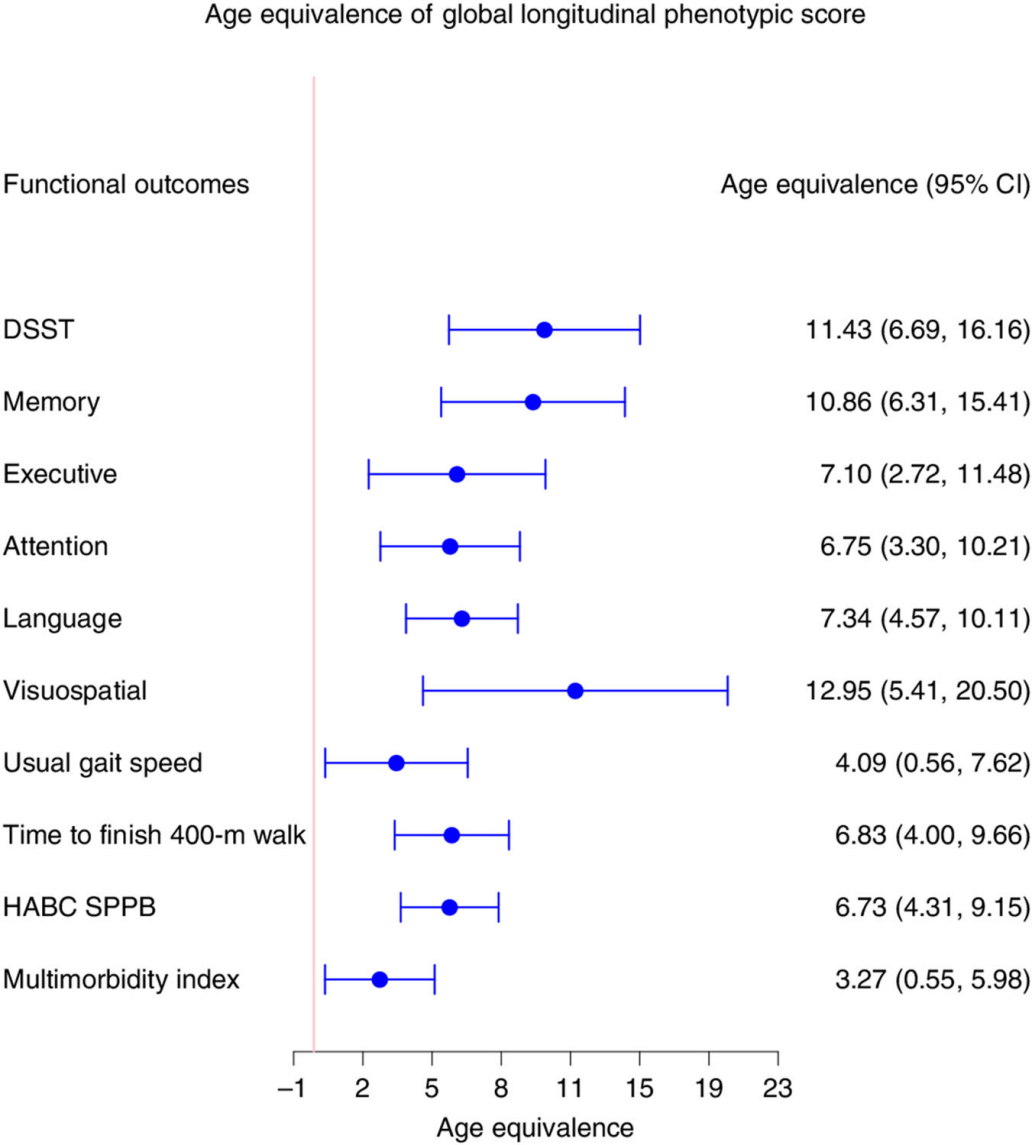
Age equivalence of a one-point difference in global longitudinal phenotypic score for different functional outcomes. Estimated age equivalence of a one-point difference in global longitudinal phenotypic score for different functional outcomes, including cognitive function, physical function and multimorbidity. Age equivalence presented here is a scaled regression coefficient (point estimates and 95% CIs) relating longitudinal phenotypic score and rate of change in functions and illustrates how many years older in functional age are individuals with one point higher in longitudinal phenotypic score. Participants: DSST, *n* = 921; memory, *n* = 922; executive function, *n* = 929; attention, *n* = 929; language, *n* = 929; visuospatial ability, *n* = 919; usual gait speed, *n* = 968; time to finish 400-m walk, *n* = 943; HABC SPPB, *n* = 968; multimorbidity index, *n* = 828); also see Supplementary Table 1b for more details.

In the BLSA, physical function was measured using usual gait speed over 6 m, time to finish a 400-m walk (measured by time to walking 400 m as quickly as possible ^25^) and the Health, Aging and Body Composition short physical performance battery (HABC SPPB; a continuous score derived from gait speed over 6 m unrestricted and over a 20-m-wide course of repeated chair stands, and sequential balance testing, with higher scores indicating better function)^26^. As shown in Fig. 4, lower global longitudinal phenotypic scores (that is, decelerated decline in aging phenotypes) were associated with slower physical function decline across all measures. Adjusting for sex, baseline age, height, weight, interactions between sex and time, and baseline age and time, a global longitudinal phenotypic score (that is, decelerated decline in aging phenotypes) one point lower was associated with (1) slower (0.0032 m s^−1^) annual decline in gait speed (95% confidence interval (CI): 0.0004, 0.0059), (2) 2.57 s less annual increase in time to finish a 400-m walk (95% CI: 1.51, 3.64) and (3) 0.0177 less annual decline in HABC SPPB score (95% CI: 0.0113, 0.0240) (Supplementary Table 2). In our study population, gait speed declined at an average annual rate of 0.014 m s^−1^, and 1 year older in chronological age was associated with faster decline in gait speed with an annual increment of 0.0008 m s^−1^ (Supplementary Tables 3 and 4). The associations between one point ‘lower’ in global longitudinal phenotypic score and physical function measures were equivalent to 4.09, 6.83 and 6.73 years ‘younger’ gait speed, time to finish a 400-m walk and HABC SPPB score, respectively (Fig. 5). Interestingly, associations between domain-specific longitudinal phenotypic scores and physical function measures were all weaker than that between global longitudinal phenotypic score and physical function measures (Supplementary Fig. 6). This finding further confirms the notion that building a robust longitudinal phenotypic score of aging requires the combination of information across different domains of measurement.

### Longitudinal phenotypic aging and change in cognitive function.

**Fig. 6 ∣:**
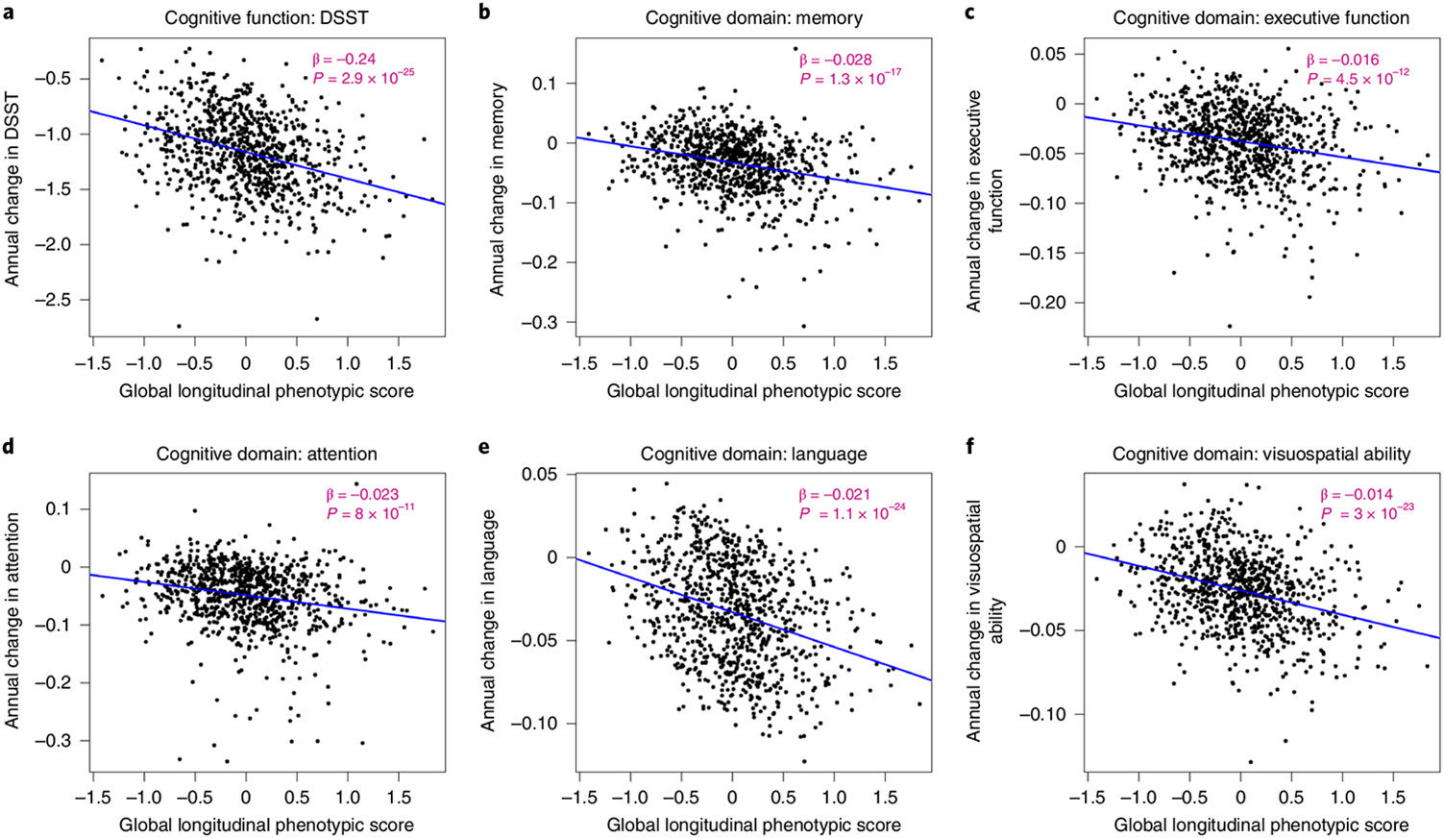
Relationship between global longitudinal phenotypic score and changes in cognitive function. **a**–**c**, Higher global longitudinal phenotypic score indicates accelerated phenotypic aging trajectories. Higher annual decreases in DSST (**a**), memory (**b**), executive function (**c**), attention (**d**), language (**e**) and visuospatial ability (**f**) indicate faster decline of cognitive function. Memory score is constructed by the average of standardized immediate recall and long-delay-free recall from the CVL test. Language score is constructed as the average of standardized letter fluency and standardized category fluency. Attention score is constructed as the average standardized log-transformed trail-making tests part A and digit span forward. Executive function is constructed by the average of standardized log-transformed trail-making tests part B and digit span backward. Visuospatial ability is calculated by standardized cart rotations test. Two-sided tests were used, and the displayed *P* values were not adjusted for multiple comparisons. Participants with at least two visits: DSST, *n* = 867; memory, *n* = 877; executive function, *n* = 894; attention, *n* = 894; language, *n* = 892; visuospatial ability, *n* = 878; also, see Supplementary Table 1b for more details.Source data

Measures of cognition used for this analysis cover memory, executive function, attention, language and visuospatial ability. The digital symbol substitution test (DSST) score was kept alone because this test covers more than one domain and has been clinically considered sensitive to change in cognition globally. As shown in Fig. 6, a higher global longitudinal phenotypic score (that is, accelerated decline in aging phenotypes) was associated with faster cognitive decline. Adjusting for baseline age, sex, race, years of education and interactions between sex and time, and baseline age and time, one point higher in global longitudinal phenotypic score (that is, accelerated decline in aging phenotypes) was associated with 0.236 faster annual decline in DSST score (95% CI: 0.138, 0.334) and with significantly faster annual decline in executive function, attention, memory, language and visuospatial ability (Supplementary Table 2). In our study population, DSST declined at an average annual rate of 1.159, while 1 year older in chronological age was associated with faster decline in DSST, with an annual increment of 0.021 (Supplementary Tables 3 and 4). One point ‘lower’ in global longitudinal phenotypic score was equivalent to 6.75–12.95 years ‘younger’ chronological age in terms of rate of cognitive decline across different measures of cognition (Fig. 5). Like the physical function analyses, associations between domain-specific longitudinal phenotypic scores and cognitive functions were all weaker than that between global longitudinal phenotypic score and cognitive test performance (Supplementary Fig. 6).

### Longitudinal phenotypic aging, multimorbidity and lifespan.

**Fig. 7 ∣:**
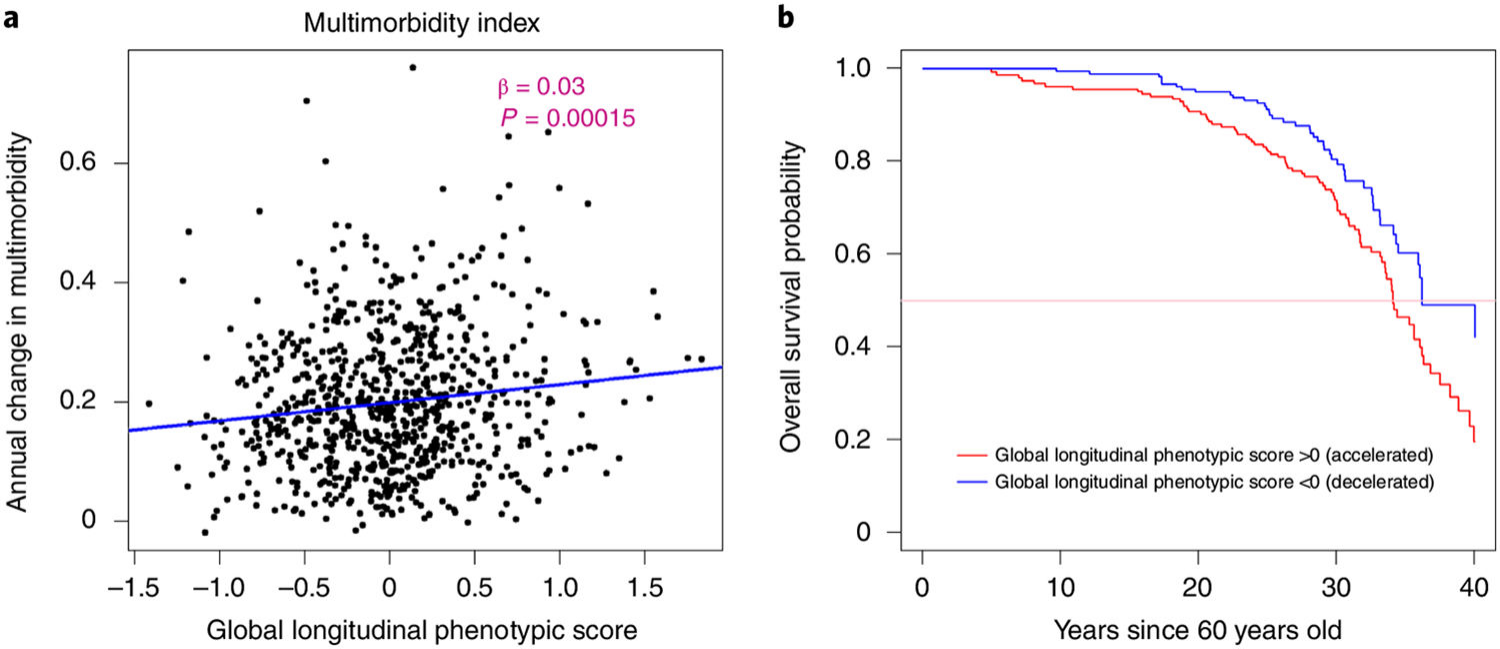
Relationship of global longitudinal phenotypic score with change of multimorbidity index and survival probability. **a**, Higher global longitudinal phenotypic score indicates accelerated phenotypic aging trajectories. Higher annual increase in multimorbidity indicates faster accumulation of chronic diseases. **b**, Among 893 participants aged >60 years during follow-up, the group with global longitudinal phenotypic score >0 (red) includes participants who experienced accelerated phenotypic aging trajectories, showing higher mortality compared with those with global longitudinal phenotypic score ≤0 (black; unadjusted time ratio (95% CI) = 0.87 (0.80, 0.95), *P* = 0.001, by fitting of Weibull distribution.) Two-sided tests were used, and the displayed *P* value was not adjusted for multiple comparisons.Source data

Under the assumption that the aging process is the major cause of chronic disease and functional impairment, the rate of accumulation of chronic disease or health/functional problems can be considered a biomarker of accelerated aging. Using a multimorbidity index previously operationalized in the BLSA as the total number of 15 candidate chronic conditions^27^, as shown in Fig. 7a, a lower global longitudinal phenotypic score (that is, decelerated decline in aging phenotypes) was associated with slower increase in the multimorbidity index. Adjusting for baseline age, sex and interactions between sex and time, and baseline age and time, one point lower in global longitudinal phenotypic score (that is, decelerated decline in aging phenotypes) was independently associated with 0.025 fewer morbidities accumulated per year (95% CI: 0.004, 0.046) (Supplementary Table 2). In our study population, the multimorbidity index increased at an average annual rate of 0.199, and 1 year older in chronological age was associated with faster increase in multimorbidity index, with an annual increment of 0.008 (Supplementary Tables 3 and 4). One point ‘lower’ in the global longitudinal phenotypic score was independently associated with change in multimorbidity at the rate equivalent to being 3.27 years ‘younger’ compared with the overall population (Fig. 5). We also examined the relationship between global longitudinal phenotypic score and mortality. Survival curves stratified by global longitudinal phenotypic score are shown in Fig. 7b. Adjusting for age, sex and education, among those surviving to at least 60 years, one point ‘higher’ in global longitudinal phenotypic score was associated with 12% shorter survival time (adjusted time ratio: 0.88, 95% CI: 0.81, 0.96) (Supplementary Fig. 7 and Supplementary Table 5).

Metrics of the rate of aging may be particularly useful in relatively young and healthy individuals, at the time when chronic disease is infrequent and traditional measures of physical function may be less informative because of substantial reserve capacity and strong resilience. To address this issue, we analyzed relationships between the global longitudinal phenotypic score and measures of physical and cognitive function within three age strata: ≤50, 51–79 and ≥80 years for physical functions and 50–65, 66–79 and ≥80 years for cognitive functions (Supplementary Figs. 8 and 9). Findings were consistent across age strata although, not surprisingly, associations appear to be stronger at older ages. We tested whether the relationship between global longitudinal phenotypic score and change in physical and cognitive functions was stronger among older adults, and significant interactions were found for usual gait speed (*P* = 0.002), time to finish a 400-m walk (*P* < 0.001), HABC SPPB (*P* < 0.001), memory (*P* < 0.001) and attention (*P* = 0.001), but not for DSST, executive function, language or visuospatial ability (Supplementary Figs. 8 and 9).

### Comparison with cross-sectional phenotypic and epigenetic summaries.

**Fig. 8 ∣:**
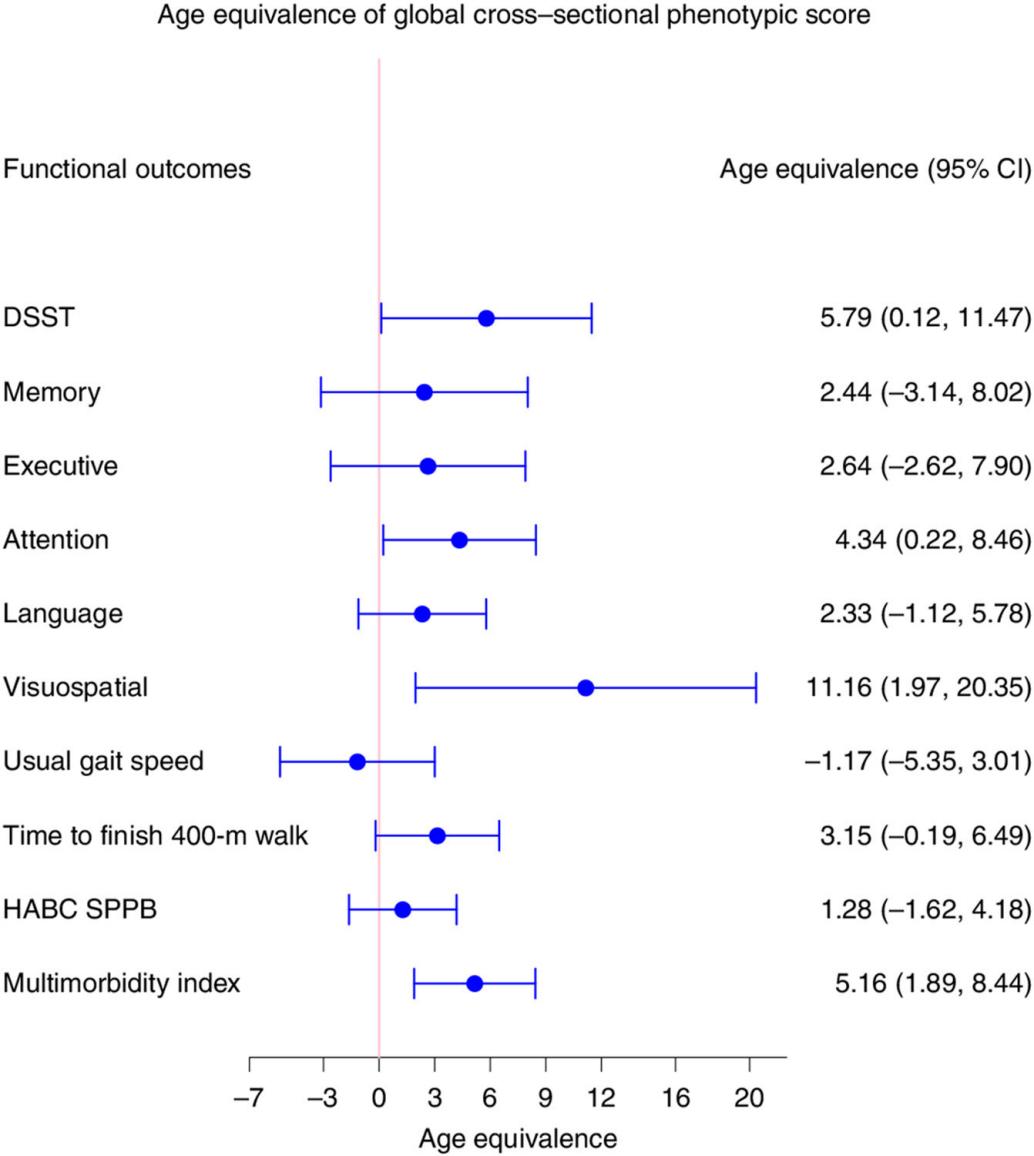
Age equivalence of a one-point difference in global cross-sectional phenotypic score for different functional outcomes. Estimated age equivalence of a one-point difference in global cross-sectional phenotypic score for different functional outcomes, including cognitive function, physical function and multimorbidity. Age equivalence is a scaled regression coefficient (point estimates and 95% CIs) relating cross-sectional phenotypic score and rate of changes in functions and illustrates how many years older in functional age are individuals with one point higher in cross-sectional phenotypic scores. Number of participants: DSST, *n* = 922; memory, *n* = 922; executive function, *n* = 929; attention, *n* = 929; language, *n* = 929; visuospatial ability, *n* = 919; usual gait speed, *n* = 968; time to finish 400-m walk, *n* = 943; HABC SPPB, *n* = 968; multimorbidity index, *n* = 828; also see Supplementary Table 1b for more details.

To further understand the added value of using longitudinal data to estimate the rate of aging, we developed a global cross-sectional phenotypic score for the four domains using the same analytical approach for estimation of longitudinal score. Specifically, we summarized the differences between measures of phenotypes in individual participants and estimated average measures of the same phenotypes in the population of the same age and sex. The associations between global cross-sectional phenotypic score and changes in physical and cognitive functions were substantially weaker than those between global longitudinal phenotypic score and changes in physical and cognitive functions, except for multimorbidity which was comparable (Fig. 8).

Epigenetic age acceleration metrics based on measures of DNA methylation are generally considered powerful biomarkers for tracking biological aging^28^. We also investigated the relationships between six popular epigenetic age acceleration measurements and changes in physical and cognitive functions in the BLSA population. In comparison with the global longitudinal phenotypic score, associations of epigenetic clocks with changes in physical and cognitive functions were much weaker, indicating that these epigenetic age clocks may provide some information on the biological age of an individual but are poor metrics of longitudinal rates of aging in a relatively healthy population (Supplementary Fig. 10).

## Discussion

Over the past decade, several studies have provided evidence that biological aging by itself is profoundly involved in the pathogenesis of many diseases and age-related health conditions^29^. On the background of genetic predispositions, nongenetic stressors (environmental, behavioral and social) show substantial stochastic variability, suggesting that they are potentially modifiable by preventive interventions^5,8,30^. Nevertheless, chronological age remains the most used proxy for aging in both clinical and research settings. Moreover, the adverse effect of older chronological age on health has not been comprehensively and exhaustively explained in epidemiological studies by the many measures of ‘biological aging’ proposed in the literature^31-33^. A critical element in the quest for robust biomarkers of biological aging is the definition of the aging phenotype^4^. Many studies have relied on chronological age as the standard of reference to calculate score-based weighted linear or nonlinear combinations of different types of ‘omics’ and used deviation from age estimators as indicative of accelerated or decelerated aging^14,34,35^. Others have relied on either mortality prediction or health predictors obtained by aggregating clinical dimensions associated with aging and/or mortality^11,13^. Overall, these approaches do not consider aging as a dynamic process or appreciate that heterogeneity between individuals in their aging trajectories substantially expands in late life^5,7^. In addition, different phenotypes of aging follow average trajectories that are characteristically linear or nonlinear, and thus cross-sectional summarization of these phenotypes may miss important information about the aging process and introduce substantial bias^6,36,37^. Indeed, in comparison with the global longitudinal phenotypic score, in our study, the global cross-sectional phenotypic score and epigenetic age accelerations showed weaker associations with changes in physical and cognitive functions.

The work presented here attempts to overcome the limitations of previous research by generating a global longitudinal phenotypic score based on summarizing the difference between individuals’ longitudinal trajectories and population average age- and sex-specific trajectories across 35 aging phenotypes from four predefined domains. We have previously defined average sex- and age-specific trajectories of these phenotypes across these same phenotypes using data from the BLSA and found some to be nonlinear and differ between men and women^6^. Here, we compared individual longitudinal trajectories with estimated population average age- and sex-specific trajectories (at the same age and sex as the individual). Then, for each individual, we summarized domain-specific scores by aggregating data within domain-specific phenotypes and, subsequently, creating a global longitudinal phenotypic score by averaging across four phenotypic domains. Using this approach, we demonstrate that participants experienced quite heterogeneous rates of phenotypic aging across a wide age range, from mid-20s to 90s, and such variability is highly informative. We have previously postulated that, independent of confounders, a score based on longitudinal changes in phenotypes would be associated with parallel decline of physical and cognitive functions^4,6,21^. Concordant with this hypothesis, we found that the domain-specific scores using longitudinal phenotypes were robustly and significantly associated with various measures of physical and cognitive function (Supplementary Fig. 6). Furthermore, those who experienced accelerated global phenotypic aging had faster decline in physical and cognitive functions, and accumulation of multiple morbidities, and experienced shorter lifespan. The associations between global longitudinal phenotypic score and changes in physical and cognitive functions remained strong across different age groups (Supplementary Figs. 8 and 9). Because the correlations between domain-specific longitudinal slopes were weak, these findings strongly suggest that combining information on rate of aging across different domains is more powerful than relying on measures in a single domain for quantification of the overall rate of phenotypic aging. When we ranked the importance of domain-specific longitudinal phenotypic scores based on their ability to explain the variability in changes of functions (Supplementary Table 6), some results were unexpected (for example, the body composition domain being ranked higher than the neuroplasticity/neurodegeneration domain for changes in attention), indicating that relationships between phenotypic and functional aging constructs are not always obvious.

Altogether, our study demonstrates that the conceptual framework based on four domains for phenotypic aging can capture aging from early adulthood to late life and that longitudinal change in phenotypes is associated with longitudinal change in multiple dimensions of function. The conceptual design and analytical approach of our study are novel in many ways. First, we present a clear conceptual framework that defines four phenotypic domains of aging that were hypothesis driven and predetermined. Second, our global score of longitudinal phenotypes was created based on longitudinal change in phenotypes, which is not biased by assumptions of linearity that do not hold for many important aging phenotypes^6,21^. Third, we included participants across a wide age range, allowing us to observe phenotypic aging from early adulthood to late life. Fourth, we had multiple longitudinal measurements of both physical and cognitive function, enabling investigation of whether those with accelerated phenotypic aging consistently experienced faster functional decline.

Most previous work aimed at quantification of phenotypic or biological aging has relied on cross-sectional data. For example, Levine et al. and Liu et al. used data from the National Health and Nutrition Examination Survey^10,38^, a study where most phenotypes were evaluated using blood-based measures and thus with limited coverage of the four phenotypic domains^10,38^. Other work attempting to quantify the rate of aging using longitudinal changes in phenotypes summarized the pace of aging using rate of change in several phenotypes, but the study population was derived from the same birth cohort with a narrow age range^12,39^. While these studies substantially contributed to our understanding of phenotypic aging, none of the proposed rate of change measures were validated against longitudinal change in physical and cognitive function and multimorbidity, which are critical outcomes in the geriatric population and the most relevant for quality of life^10,12,39^.

To facilitate interpretation of our findings, we estimated the age equivalence of a single point change in global longitudinal phenotypic score in our study population. One point higher in global longitudinal phenotypic score (that is, accelerated decline in aging phenotypes) was equivalent to an additional 4–7 years in chronological age across physical function measures and to an additional 7–13 years in chronological years across cognitive function measures. These results have three implications.

First, the global longitudinal phenotypic score captured aspects of phenotypic aging that are relevant to age-associated health and functional changes independent of age, sex and other time-invariant confounders. The consistent association with a wide range of measures of physical and cognitive function highlights the robustness of our summarized score. Second, the magnitude of age equivalents differed slightly across different cognitive and physical measurements. This not only reflects differential rates of change in physical and cognitive functional assessments but also implies that if optimization of the phenotypic age is desired, it must be function specific and perhaps measurement specific. Third, when we compared the age equivalence of domain-specific longitudinal phenotypic scores with that of global longitudinal phenotypic score for physical and cognitive function, we found the global score showed the largest age-equivalent power, meaning that the combined information contributed by all four domains (body composition, energetics, homeostatic mechanism and neuroplasticity/neurodegeneration) is more informative than each individual domain separately.

Some aspects of our findings deserve consideration. This work has several important implications. First, our metric captures aspects of phenotypic aging apparent in early adulthood and within a relatively healthy population. Second, a conceptual framework that views phenotypic aging across multiple domains is essential to capturing a multifaceted picture of aging. Third, the hierarchical and temporal relationships between biological, phenotypic and functional aging provide a powerful framework to study the aging process, and application of this robust framework better facilitates identification of mechanistic biomarkers that underlie the aging process and pace of aging in specific individuals. Consistent with our previously stated hypothesis, in the future, it will be important to identify longitudinal changes in omics biomarkers associated with antecedent and parallel changes in phenotypic aging and, in turn, functional aging^4,6,21^.

Despite the many positive features of our study, several limitations should be noted. First, the comprehensive longitudinal, state-of-the-art measurements of phenotypes in the BLSA are costly in terms of both time and money, so it will be challenging to find another independent cohort with the same or similar set of measurements to exactly replicate our results. Nevertheless, our conceptual framework can be easily adapted to a smaller collection of phenotypes representative of the four domains provided a good number of longitudinal observations is available. In addition, one primary aim of developing a global longitudinal phenotypic score is to derive a ‘biological aging clock’ using a longitudinal approach. Once biological aging clocks are developed, they can be validated across multiple studies and compared with other clocks developed using more conventional cross-sectional measures. Second, the relatively modest sample size of our study population has limited some aspects of our statistical analyses. For example, because of this limitation, we empirically put equal weights on all four domains rather than optimizing the weights using computation-intensive algorithms to avoid overfitting ^40^. Our analysis showed strong associations between global longitudinal phenotypic score and changes in physical and cognitive function. We did not have sufficient observations to fully separate these two dimensions over time, which would have strengthened the assumption of causality. However, there is no guarantee that a fully lagged analysis would work because individuals on a certain trajectory may change trajectory over time as the rate of aging is continuously modulated by many factors. Third, although we attempted to collect these phenotypes as comprehensively as possible, we might not have all of the relevant phenotypic measures (for example, more detailed brain measures^40^ and immune profiles ^35^) for all participants. Nevertheless, despite these potential limitations, application of our conceptual framework produced robust and convincing results.

There are several strengths in this work that outweigh its limitations. Indeed, the longitudinal trajectories of aging phenotypes have been examined one by one in detail in our previous work, which helped us refine and validate the conceptual framework for this study^6,21^. The longitudinal data enabled us to focus on the aging effect and control for the time-invariant unmeasured confounding (for example, early life exposures) using mixed-effect models^37^. In regard to the calculation of global longitudinal phenotypic score, we adopted a flexible strategy to include everyone with longitudinal information on at least one phenotype in each of four domains. This operationalization makes the creation of such a score feasible in other cohorts in the future. One important aspect of this approach is that it can discriminate between cohort effects that are already present from birth and aging effects (true differences in trajectories that occur over the lifespan). The framework illustrated here can be easily adopted in other cohorts if longitudinal data in phenotypes covering four phenotypic domains are collected. Linking this global longitudinal phenotypic score with cellular and molecular measurements will improve the potential to identify the underlying biological mechanisms of aging.

In conclusion, this work demonstrates the usefulness of our hierarchical and temporal conceptual framework regarding three metrics of aging. Our work proposes a powerful method to summarize the longitudinal trajectory of aging phenotypes. These results can build the foundation for both research focused on understanding the biology of aging and the implementation of phenotypic aging measurement in trials targeting the rate of aging and its consequences.

## Methods

### Study design and study participants.

The BLSA study protocol has been approved by the Internal Review Board of the Intramural Research Program of the National Institutes of Health; participants provided written informed consent after receiving a comprehensive description of the study procedures, including possible risks. The BLSA, a study of normative human aging, was established in 1958, comprehensively revised in 2003 with more extensive domain-based phenotypic measurements and is conducted by the National Institute on Aging Intramural Research Program^6^. All participants are community-dwelling volunteers free of major chronic conditions upon enrollment. Detailed inclusion/exclusion criteria are described in our previous work^6^. To acknowledge the faster functional decline in the later part of life, enrolled participants are followed up with an age-dependent frequency (<60 years every 4 years, 60–79 years every 2 years, ≥80 years every year)^6^. The analytic sample for the current study mainly consists of participants who underwent repeated phenotypic measurements during their clinic visits between January 2005 and December 2019. All assessments were collected by trained and certified technicians following standardized protocols. Because the BLSA is an observational cohort study, no blinding was used.

### Measurements.

#### Measurement of aging phenotypes.

Since revision in 2003, the phenotypes measured in the BLSA have been aimed at capturing four phenotypic domains that conceptually serve as bridges between geroscience—which focuses on the cellular and molecular mechanisms of aging—and gerontology and geriatrics, which concentrate on age-related diseases and functional decline^21^. Namely, these four phenotypic domains are body composition, energetics, homeostatic mechanisms and neuroplasticity/neurodegeneration. Based on our conceptual framework, the measures of these four domains are considered as the ‘phenotypic clusters of aging’, which are presumed to be the phenotypic manifestations of underlying biological aging conceptualized as the ratio between damage and repair at molecular/cellular levels^21^. The aging phenotypes collected here have been examined longitudinally in our previous work^6^. Phenotypes representing body composition include body mass index, waist circumference, waist-to-height ratio, total lean mass, appendicular lean mass, total fat mass and mid-thigh area. Phenotypes covering the energetics domain include parameters of energy availability and energy consumption as measured by oxygen consumption, and cardiorespiratory fitness. Phenotypes used to present homeostatic mechanisms include inflammation markers, fasting glucose, lipid profile, blood pressure, carotid/femoral pulse wave velocity and 24-h creatinine clearance. Phenotypes used to represent neurodegeneration/neuroplasticity domain cover both the central and peripheral nervous systems. The central nervous system was assessed by brain volumes (total brain, white matter, gray matter and ventricular), and the peripheral nervous system by nerve conduction velocity.

### Measurements of functional aging.

#### Physical functions.

In the BLSA, physical function was measured with usual gait speed over 6 m, time to finish a 400-m walk (measured by time required to walk 400 m as quickly as possible^15^) and the HABC SPPB^25,26^.

#### Cognitive functions.

Global cognitive function was measured using DSST. Domain-specific cognition (memory, executive function, attention, language and visuospatial ability) are derived from trails-making tests A and B, digits forward and digits backward, the California verbal learning (CVL) test, letter and category and the card rotations test.

#### Multimorbidity index and mortality.

The multimorbidity index comprised age-related chronic conditions. Vital status was determined using telephone follow-up, correspondence and searches of the National Death Index.

### Statistical analysis.

#### Creation of global and domain-specific longitudinal phenotypic scores.

Step 1: For each phenotype, quantile normalization was used to account for the different measurement methods^41^. To calculate the difference between an individual’s rate of change and estimated sex- and age-specific rate of change, we used the linear mixed model with random intercept and random slope (as below) for male and female separately. In this model, *b*_*i*_ is then extracted and used as the difference between an individual’s rate of change and estimated sex- and age-specific rate of change.

With *a*_i_ denoting random intercept and *b*_i_ denoting random slope, the main function we fit is in the following form:

$${\text{Phenotype}}_{ij}=\alpha \;({\text{cov}}_{i})+a_{i}+(\beta \;({\text{cov}}_{i})+b_{i})\times t_{ij}+e_{ij}\;\text{for subject}\;i\;\text{at time}\;j,$$

where (*a*_*i*_, *b*_*i*_ ≈ *N*(0,*G*), *e*_*ij*_ ≈ *N*(0, *σ*^2^ *R*), *α*(cov_*i*_) is a function of covariates described above and *β*(cov_i_) is a polynomial function of baseline_age_*i*_.

Further, the difference between an individual’s rate of change and sex- and age-specific population rate of change (that is, the random intercept) was standardized (to mean = 0 and s.d. = 1) and transformed to −3, −2, −1, 0, 1, 2, 3, termed ‘individual phenotype-specific score’ (3, 2 and 1 corresponding, respectively, to 2.5–5.0, 1.5–2.5 and 0.5–1.5 s.d. faster/accelerated decline in phenotypes; 0 corresponding to within 0.5 s.d. of changes in phenotypes; −1, −2 and −3 corresponding, respectively, to 0.5–1.5, 1.5–2.0 and 2.5–5.0 s.d. slower/decelerated decline in phenotypes). Figure 3 shows a conceptual illustration of accelerated and decelerated aging.

Step 2: For each domain, we calculated the domain-specific longitudinal phenotypic scores for each individual by averaging the available individual phenotype-specific score for phenotypes within each domain, followed by quantile normalization.

Step 3: The global longitudinal phenotypic score was then summarized by averaging the four domain-specific longitudinal phenotypic scores, for those with all four domain-specific scores available.

#### Examining the relationship between longitudinal phenotypic score(s) and functional outcomes/mortality.

To estimate the relationship between global and domain-specific longitudinal phenotypic score and rate of functional decline and changes in multimorbidity, linear mixed models with random intercept and random slope were used. Baseline age was defined as age at first analytic visit. For cognitive function, the models included sex, baseline age, years of education, race, longitudinal phenotypic aging score, time, baseline age × time, sex × time and longitudinal phenotypic aging score × time. For physical function, the models included sex, baseline age, baseline age squared, height, weight, longitudinal phenotypic aging score, time, baseline age × time, sex × time and longitudinal phenotypic aging score × time. For multimorbidity index, the models included sex, baseline age, baseline age squared, longitudinal phenotypic aging score, time, baseline age × time, sex × time and longitudinal phenotypic aging score × time. Because the scales of cognitive functions, physical functions and multimorbidities are different, to improve the interpretability of results, we translated the results as age equivalent, which can be interpreted as the equivalent effect of the number of years of chronological age increase on rate of changes per one point higher in global/domain-specific longitudinal phenotypic score(s)^42^. To visualize the data, we plotted the scatterplot between summarized global score and slopes of changes in cognitive functions, physical functions and multimorbidity index.

To understand the relationship between the global longitudinal phenotypic score and mortality, we used survival analysis with a semiparametric Cox model and parametric Weibull distribution to quantify the relationship between summarized global score and mortality risk, using age starting from 60 years as the timescale with adjustment for age, sex and education. Point estimates with 95% CI are reported.

#### Evaluation of the association between cross-sectional measurements and changes in physical and cognitive functions.

To understand the potential difference between the cross-sectional aging summary and our global longitudinal phenotypic scores, we also computed the association between cross-sectional phenotypic score, six epigenetic age acceleration measurements and changes in physical and cognitive functions.

Detailed information about measurements and statistical analysis is reported in Supplementary Methods, including Supplementary Methods I—Measurements for Aging Phenotypes, Supplementary Methods II—Measurements for Functional Outcomes and Supplementary Methods III—Statistical Analysis.

## Supplementary Material

Supplementary Information

Supplementary Tables

## Figures and Tables

**Fig. 1 ∣:**
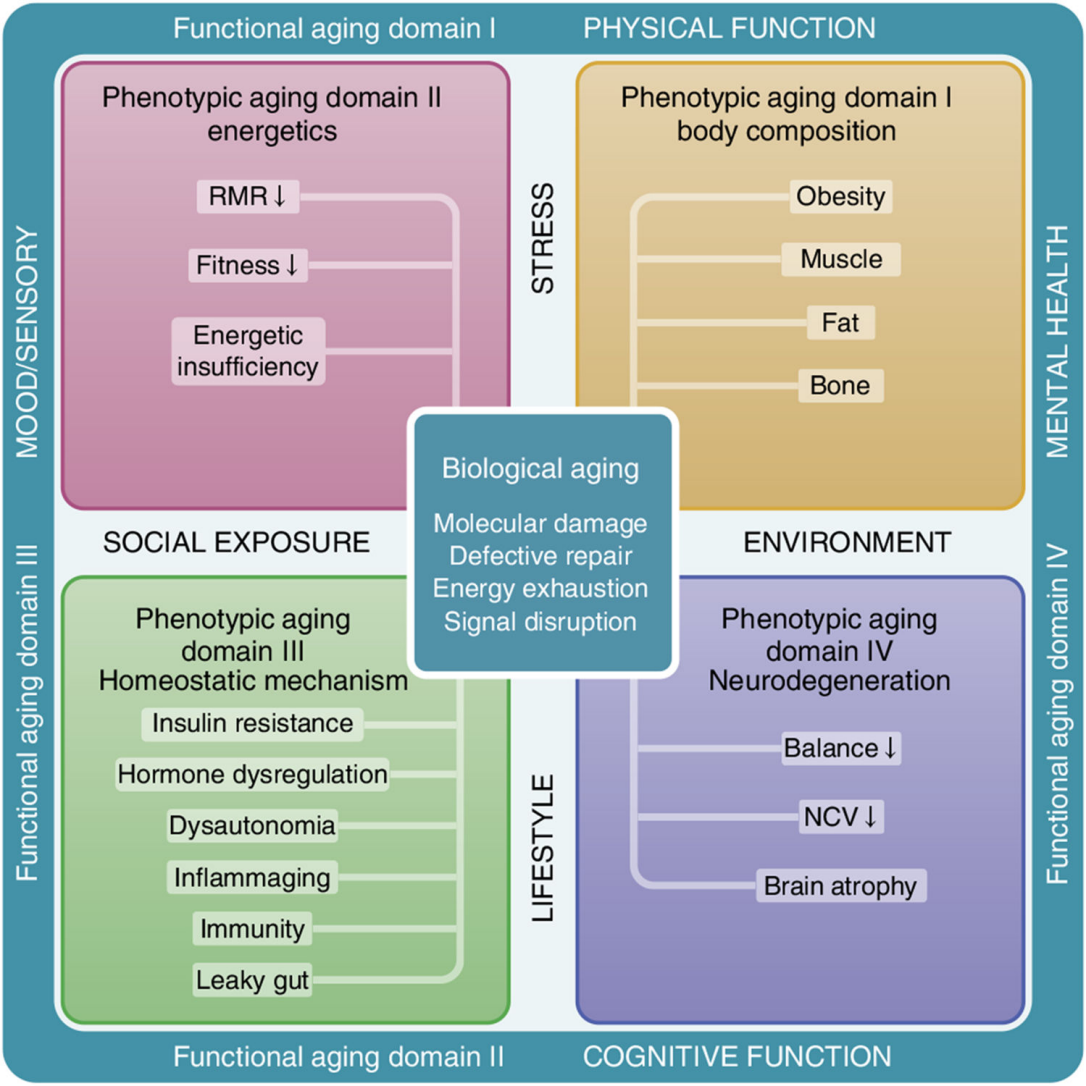
Conceptual framework underpinning the study design. Conceptual framework of three hierarchical and temporal metrics of aging—biological, phenotypic and functional. We hypothesize that biological mechanisms (center square) drive changes in aging phenotypes, which eventually lead to deterioration at functional levels (outer rim). Four phenotypic domains are proposed for measurement of aging phenotypes—body composition, energetics, homeostatic mechanisms and neuroplasticity/neurodegeneration. Examples of aging phenotypes are listed in the colored boxes. RMR, resting metabolic rate; NCV, nerve conduction velocity.

## Data Availability

The BLSA data are available upon request. Applications should be made through the website https://www.blsa.nih.gov/.

## Code availability

Complementary R code is provided in the Supplementary Material.
