# Supplementary material for: Longitudinal phenotypic aging metrics in the Baltimore Longitudinal Study of Aging
Source: Nat Aging. 2022 Jul 18;2(7):635–43. doi: 10.1038/s43587-022-00243-7 (PMC9997119; doi:10.1038/s43587-022-00243-7)
Supplement: Supplementary file 1 — Supplementary Methods I–III, Figs. 1–12 and complementary code. [file 43587_2022_243_MOESM1_ESM.pdf]

nature portfolio

https://doi.org/10.1038/s43587-022-00243-7

Supplementary information

Longitudinal phenotypic aging metrics in the Baltimore Longitudinal Study of Aging

In the format provided by the authors and unedited

Supplemental Method I – Measurements for Aging Phenotypes

The phenotypic variables used for the analysis presented in this manuscript were collected in BLSA participants during a two-/three-day clinical visit and the National Institute on Aging Clinical Research Unit. Descriptions below are consistent with those previously reported[1], and the cross-sectional correlation structure of these 35 phenotypes at baseline are shown in the Supplemental Figure 11.

Body composition domain

Changes in body composition are evident across the life span. Traditional body size measures are collected in BLSA, including waist circumference as well as weight and height, used to estimate body mass index (BMI) [2]. Anthropometric measurements were made using a standardized protocol: participants are assessed in light clothing, waist circumference was measured by tape measure, height and weight were assessed using a stadiometer and calibrated scale, respectively. Total lean mass, appendicular lean mass and total fat mass were assessed using total body dual-energy X-ray absorptiometry (DEXA; Prodigy Scanner, GE, Madison, WI) with Encore Software. While total lean mass is composed of both muscle and visceral organs, appendicular lean mass (both arms and legs) is primarily muscle mass [3]. DEXA measures are complemented with computerized tomography cross-sectional images (10 mm) at the mid-thigh area (10mm, Somatom Sensation 10, Siemens, Malvern PA) quantified using the Geanie 2.1 software (BonAlyse Oy, Jyvaskla, Finland) and Tibia Estimation Tool (TibEsT v.1.4, Makrogiannis, NIH)[4] .

Energetic domain

Both parameters of energy availability and energy consumption change with aging. In the BLSA, we collect information on oxygen consumptions (VO_2) from resting to maximal exertion. Resting metabolic rate (kcals/day), the minimal amount of energy required for living, was assessed by indirect calorimetry [5,6], using a Cosmed k4b2 portable metabolic analyzer (Cosmed, Rome, Italy) after awakening in the morning in a quiet, thermo-neutral environment, in a fasted, rested state [7]. Peak VO_2 (ml/kg/min) was assessed during a modified Balke protocol maximal treadmill test as a proxy measure of maximal energy availability (VO_2 max) [8]. The balance between energy availability and demand for physical functioning was estimated by a ratio of the energy cost of slow walking to peak walking capacity ("cost-capacity ratio") [9-11]. The energetic cost of slow walking (ml/kg/min) was assessed via indirect calorimetry (Medical Graphics Corp, St Paul, MN) during 5 minutes of slow treadmill walking at 0.67 m/sec (1.5 mph), zero percent slope [9]. Peak walking capacity was assessed during a 400 meter walk test performed in an uncarpeted corridor with the participant wearing a portable metabolic analyzer, the Cosmed K_4b^2 (Cosmed, Rome, Italy) [7,9]. Forced vital capacity (FVC) and the forced expiratory volume in the first second (FEV_1), indicators of respiratory capacity and functions, were measured using a MedGraphics Gas Exchange System (Medical Graphics Corp, St Paul, MN) through closed-circuit breath collection [12].

Homeostatic mechanisms domain

A stable homeostatic equilibrium is essential for healthy life. Some homeostatic biomarkers that are particularly relevant for the study of aging and can be measured "in vivo" in a clinical study are assessed in the BLSA. They include: chronic inflammation, insulin sensitivity/resistance, cardiovascular parameters, circulating lipids, and renal function. As biomarkers of chronic inflammation, we considered Interleukin-6 (IL-6), C-reactive protein, albumin, hemoglobin, red cell distribution width and neutrophil count. IL-6 was measured by commercial ELISA kits (R&D System, Minneapolis, MN, USA), and C-reactive protein (CRP), by ELISA (ALPCO Diagnostics, Salem NH, or Alpha Diagnostic International, San Antonio, TX or Immundiagnostik AG) [4,13]. Albumin was measured using dye binding BCG [14,15]. Hemoglobin, red blood cell width (RDW), and absolute neutrophil counts were measured by Sysmex's multiple methods[15]. Fasting plasma glucose was measured in the morning after at least 10 hours overnight fast [4,16,17]. Cardiovascular parameters included blood pressures and pulse-wave velocity. Blood pressures were measured in the supine position [18-20]. Carotid-femoral pulse wave velocity was measured using either Transcutaneous Doppler probes (model 810A, 9 to 10- Mhz probes, Parks Medical Electronics, Inc., Aloha, Oregon) or Complior SP device (Artech Medical, Paris, France) or SphygmoCor system (AtCor Medical, Sydney, Australia) by well-trained technicians [18]. Concentrations of plasma triglycerides and total cholesterol were determined by an enzymatic method (ABA-200 ATC Biochromatic Analyzer; Abbott Laboratories, Irving, TX) [21,22] and the concentration of high-density lipoprotein (HDL) cholesterol was determined by a dextran sulfate–magnesium precipitation [21,22]. Renal function was measured by body surface area adjusted 24-hour creatinine clearance, derived from 24-hour urine collection [23]. Other widely used estimation of glomerular filtration rate (ex: Modification of Diet in Renal Disease Study (MDRD), and Chronic Kidney Disease Epidemiology Collaboration (CKD-EPI)) were avoided because they include an "age" parameter [23]. Creatinine concentrations in serum and urine were measured either by the enzymatic Vitros CREA method performed on the Ortho Fusion 5.1 Analyzer (Ortho-Clinical Diagnostics, Rochester, NY), or the isotope dilution mass spectrometry (IDMS)-traceable serum creatinine assay [23].

Neurodegeneration/Neuroplasticity domain

Phenotypes used to represent neurodegeneration/neuroplasticity domain cover both the central nervous system and peripheral nervous system. The central nervous system was assessed by brain volumes (total brain volume, white matter volume, grey matter volume, and ventricular volume), and the peripheral nervous system was assessed by nerve conduction velocity. Brain volumes were measured using a 3T Philips Achieva Magnetic Resonance Imaging (MRI) system to acquire magnetization-prepared rapid gradient echo (MPRAGE) scans (repetition time =6.8ms, echo time =3.2ms, flip angle=8°, image matrix=256×256, 170 slices, pixel size=1×1mm, slice thickness=1.2mm; sagittal acquisition). Anatomical labels and global and regional brain volumes were obtained using Multi-atlas region Segmentation using Ensembles of registration algorithms and parameters (MUSE) [24]. To measure the fibular nerve conduction velocity, a trained technician performed a standard nerve conduction velocity test on the peroneal nerve. To measure the fibular nerve conduction velocity, a trained technician performed a standard nerve conduction velocity test on the peroneal nerve [25].

Reference for Supplemental Method I – Measurements for Aging Phenotypes

1 Kuo, P. L. *et al.* A roadmap to build a phenotypic metric of ageing: insights from the Baltimore Longitudinal Study of Aging. *J Intern Med* **287**, 373-394, doi:10.1111/joim.13024 (2020).

2 Fabbri, E. *et al.* Early body composition, but not body mass, is associated with future accelerated decline in muscle quality. *J Cachexia Sarcopenia Muscle* **8**, 490-499, doi:10.1002/jcsm.12183 (2017).

3 Chiles Shaffer, N. *et al.* Muscle Quality, Strength, and Lower Extremity Physical Performance in the Baltimore Longitudinal Study of Aging. *J Frailty Aging* **6**, 183-187, doi:10.14283/jfa.2017.24 (2017).

4 Moore, A. Z. *et al.* Difference in muscle quality over the adult life span and biological correlates in the Baltimore Longitudinal Study of Aging. *J Am Geriatr Soc* **62**, 230-236, doi:10.1111/jgs.12653 (2014).

5 McAnena, O. J., Harvey, L. P., Katzeff, H. L. & Daly, J. M. Indirect calorimetry: comparison of hood and mask systems for measuring resting energy expenditure in healthy volunteers. *JPEN J Parenter Enteral Nutr* **10**, 555-557, doi:10.1177/0148607186010006555 (1986).

6 Rumpler, W. V., Seale, J. L., Conway, J. M. & Moe, P. W. Repeatability of 24-h energy expenditure measurements in humans by indirect calorimetry. *Am J Clin Nutr* **51**, 147-152, doi:10.1093/ajcn/51.2.147 (1990).

7 Schrack, J. A., Simonsick, E. M. & Ferrucci, L. Comparison of the Cosmed K4b(2) portable metabolic system in measuring steady-state walking energy expenditure. *PLoS One* **5**, e9292, doi:10.1371/journal.pone.0009292 (2010).

8 Fleg, J. L. *et al.* Accelerated longitudinal decline of aerobic capacity in healthy older adults. *Circulation* **112**, 674-682, doi:10.1161/CIRCULATIONAHA.105.545459 (2005).

9 Schrack, J. A., Simonsick, E. M. & Ferrucci, L. The energetic pathway to mobility loss: an emerging new framework for longitudinal studies on aging. *J Am Geriatr Soc* **58 Suppl 2**, S329-336, doi:10.1111/j.1532-5415.2010.02913.x (2010).

10 Schrack, J. A., Simonsick, E. M. & Ferrucci, L. The relationship of the energetic cost of slow walking and peak energy expenditure to gait speed in mid-to-late life. *Am J Phys Med Rehabil* **92**, 28-35, doi:10.1097/PHM.0b013e3182644165 (2013).

11 Schrack, J. A., Simonsick, E. M., Chaves, P. H. & Ferrucci, L. The role of energetic cost in the age-related slowing of gait speed. *J Am Geriatr Soc* **60**, 1811-1816, doi:10.1111/j.1532-5415.2012.04153.x (2012).

12 Vaz Fragoso, C. A. *et al.* Aging-Related Considerations When Evaluating the Forced Expiratory Volume in 1 Second (FEV1) Over Time. *J Gerontol A Biol Sci Med Sci* **71**, 929-934, doi:10.1093/gerona/glv201 (2016).

13 Warren, K. N. *et al.* Elevated Markers of Inflammation Are Associated With Longitudinal Changes in Brain Function in Older Adults. *J Gerontol A Biol Sci Med Sci* **73**, 770-778, doi:10.1093/gerona/glx199 (2018).

14 Giallauria, F. *et al.* Arterial stiffness and vitamin D levels: the Baltimore longitudinal study of aging. *J Clin Endocrinol Metab* **97**, 3717-3723, doi:10.1210/jc.2012-1584 (2012).

15 Schrack, J. A., Knuth, N. D., Simonsick, E. M. & Ferrucci, L. "IDEAL" aging is associated with lower resting metabolic rate: the Baltimore Longitudinal Study of Aging. *J Am Geriatr Soc* **62**, 667-672, doi:10.1111/jgs.12740 (2014).

16 Chia, C. W. *et al.* GIP contributes to islet trihormonal abnormalities in type 2 diabetes. *J Clin Endocrinol Metab* **99**, 2477-2485, doi:10.1210/jc.2013-3994 (2014).

17 Chia, C. W. *et al.* Regular low-calorie sweetener consumption is associated with increased secretion of glucose-dependent insulinotropic polypeptide. *Diabetes Obes Metab* **20**, 2282-2285, doi:10.1111/dom.13328 (2018).

18 AlGhatrif, M. *et al.* Longitudinal trajectories of arterial stiffness and the role of blood pressure: the Baltimore Longitudinal Study of Aging. *Hypertension* **62**, 934-941, doi:10.1161/HYPERTENSIONAHA.113.01445 (2013).
19 Najjar, S. S. *et al.* Pulse wave velocity is an independent predictor of the longitudinal increase in systolic blood pressure and of incident hypertension in the Baltimore Longitudinal Study of Aging. *J Am Coll Cardiol* **51**, 1377-1383, doi:10.1016/j.jacc.2007.10.065 (2008).
20 Pearson, J. D., Morrell, C. H., Brant, L. J., Landis, P. K. & Fleg, J. L. Age-associated changes in blood pressure in a longitudinal study of healthy men and women. *J Gerontol A Biol Sci Med Sci* **52**, M177-183, doi:10.1093/gerona/52a.3.m177 (1997).
21 Semba, R. D. *et al.* Altered Plasma Amino Acids and Lipids Associated With Abnormal Glucose Metabolism and Insulin Resistance in Older Adults. *J Clin Endocrinol Metab* **103**, 3331-3339, doi:10.1210/jc.2018-00480 (2018).
22 Tanaka, T. *et al.* Plasma proteomic signature of age in healthy humans. *Aging Cell* **17**, e12799, doi:10.1111/acel.12799 (2018).
23 Dowling, T. C., Wang, E. S., Ferrucci, L. & Sorkin, J. D. Glomerular filtration rate equations overestimate creatinine clearance in older individuals enrolled in the Baltimore Longitudinal Study on Aging: impact on renal drug dosing. *Pharmacotherapy* **33**, 912-921, doi:10.1002/phar.1282 (2013).
24 Doshi, J. *et al.* MUSE: MUlti-atlas region Segmentation utilizing Ensembles of registration algorithms and parameters, and locally optimal atlas selection. *Neuroimage* **127**, 186-195, doi:10.1016/j.neuroimage.2015.11.073 (2016).
25 Deshpande, N., Metter, E. J., Ling, S., Conwit, R. & Ferrucci, L. Physiological correlates of age-related decline in vibrotactile sensitivity. *Neurobiol Aging* **29**, 765-773, doi:10.1016/j.neurobiolaging.2006.12.002 (2008).

Supplemental Method II – Measurements of Functional Outcomes

Physical Functions

In BLSA, physical function was measured using usual gait speed over 6 meters, time to walk 400 meters as quickly as possible (1), and the Health Aging, and Body Composition short physical performance battery (HABC SPPB) (a continuous score derived from gait speed, chair stand, and balance test, with higher scores indicating better function) (1, 2). For the measurement of usual gait speed, BLSA participants stood with their feet behind a taped starting line and were asked to walk at a "normal comfortable pace" over a course of 6m. For the measurement of endurance walking, BLSA participants completed a self-paced endurance walk test over 400m as fast as possible, which was performed over a 20-m course (1).

Cognitive Functions

Several cognitive tests were administered to assess cognition in the BLSA (3). These tests included digit symbol substitution test (DSST), the Digit Span Forward and Backward subtest of the Wechsler Adult Intelligence Scale – Revised (WAIS-R) (4), time needed to completed the Trail Making Tests (TMT) Part A & B (5), number of correct words recalled in immediate recall and long-delay free recall (20 – 30 minutes) from the California Verbal Learning Test (CVLT) (6), letter fluency (7), category fluency (8), and card rotations tests (3, 9, 10). DSST reflected the multiple cognitive domains. Trails Making Tests A & B, Digits Forward, and Digits Backward were used to capture executive function and attention. CVLT was used to assess the memory ability. Letter and category fluency tests were used to measure language ability. Card Rotations test was used to capture visuospatial abilities of participants.

As to the analysis, we kept the DSST (Digit Symbol Substitution Test) aside because it is thought to involve more than one domain. A memory score was constructed as the average of standardized immediate recall and long-delay free recall from California Verbal Learning test. A language score was constructed as the average of standardized letter fluency and standardized category fluency scores. An attention score was constructed as the average of standardized log-transformed Trail Making Tests Part A and Digit Span Forward scores. An executive function function score was constructed as the average of the standardized log-transformed Trail Making Tests Part B and Digit Span Backward. Visuospatial ability is calculated by standardized Cart Rotations test.

Multimorbidity Index and Mortality

Multimorbidity is defined as the condition of simultaneous occurrence of two or more diseases. Multimorbidity was assessed at each visit as number of diagnosed chronic diseases from a predefined list. The conditions include hypertension, diabetes mellitus, coronary artery disease, congestive heart failure, stroke, chronic obstructive pulmonary disease, chronic kidney disease,

cognitive decline, cancer, anemia, Parkinson's disease, Peripheral Arterial Disease, history of hip fracture, and lower extremity joint disease. The multimorbidity index was computed as the number of these conditions that were adjudicated based on pre-defined criteria (11). Vital status was determined using telephone follow-up, correspondence, and searches of the National Death Index.

Reference for Supplemental Method II – Measurements of Functional Outcomes

1. Simonsick EM, Newman AB, Visser M, Goodpaster B, Kritchevsky SB, Rubin S, et al. Mobility limitation in self-described well-functioning older adults: importance of endurance walk testing. J Gerontol A Biol Sci Med Sci. 2008;63(8):841-7.
2. Simonsick EM, Newman AB, Nevitt MC, Kritchevsky SB, Ferrucci L, Guralnik JM, et al. Measuring higher level physical function in well-functioning older adults: expanding familiar approaches in the Health ABC study. J Gerontol A Biol Sci Med Sci. 2001;56(10):M644-9.
3. McCarrey AC, An Y, Kitner-Triolo MH, Ferrucci L, Resnick SM. Sex differences in cognitive trajectories in clinically normal older adults. Psychol Aging. 2016;31(2):166-75.
4. Wechsler D. Wechsler Adult Intelligence Scale-Revised. Vol. 1.: New York: Psychological Corporation; 1981.
5. Reitan R. Trail Making Test: Manual for administration and scoring. : Reitan Neuropsychology Laboratory; 1992.
6. Delis D, Kramer J, Kaplan E, B O. California verbal learning test, research edition. : New York: The Psychological Corporation.; 1987.
7. Benton AL. Differential Behavioral Effects in Frontal Lobe Disease. Neuropsychologia. 1968;6(1):53-&.
8. Newcombe F. Missile wounds of the brain: A study of psychological deficits. Oxford, England: Oxford U. Press; 1969. vi, 145-vi, p.
9. Ekstrom R, French J, Harman H, Dermen D. Manual for kit of factor referenced cognitive tests.: Educational Testing Service; 1976.
10. Wilson JR, De Fries JC, McClearn GE, Vanderberg SG, Johnson RC, Rashad MN. Cognitive abilities: use of family data as a control to assess sex and age differences in two ethnic groups. Int J Aging Hum Dev. 1975;6(3):261-76.
11. Fabbri E, Zoli M, Gonzalez-Freire M, Salive ME, Studenski SA, Ferrucci L. Aging and Multimorbidity: New Tasks, Priorities, and Frontiers for Integrated Gerontological and Clinical Research. J Am Med Dir Assoc. 2015;16(8):640-7.

Supplemental Method III - Statistical Analysis

Part 1: Creating global and domain-specific longitudinal phenotypic scores

Three steps were used to calculate the global longitudinal phenotypic score.

Step 1: For each phenotype, quantile normalization was used to account for the different units of measure (1). Sex-specific mixed effects models of the normalized phenotypes, with random intercept, random slope, and time since first analytic visit as timescale, were fit to calculate the difference between individual's rate of change and estimated sex-and age-specific rate of change in the overall study population. Details on how the rate of changes in each phenotype were modelled are as follows. For analyses within the body composition domain, body height was included as covariates to provide an index of body size. For the energetics domain, body composition measures (fat mass, lean mass) were included as covariates in analyses concerning resting metabolic rate, peak oxygen consumption (during treadmill test and during 400m walk), and cost-capacity ratio. Height was included as a covariate for analyses concerning forced expiratory volume in the first second (FEV1), forced vital capacity (FVC), and FEV1/FVC. For the neurodegeneration/neuroplasticity domain, intracranial volume was included as a covariate in analyses concerning brain volume measurements, and the analysis was limited to those >= 40 years old (due to limited data below this age). Models with and without additional terms (baseline age squared, time X baseline age, time X baseline age squared) were performed to account for the nonlinear baseline age trend.

To calculate the difference between individual's rate of change and estimated sex- and age-specific rate of change in the population, we used the linear mixed model with random intercept and random slope (as following) for male and female separately. In such model, b_i is then extracted, and used as the difference between individual's rate of change and estimated sex-and-age specific rate of change. Below, we report the notations for the analysis described. The age-trajectories estimated for each phenotype, separately for men and women are shown in supplemental figures 2 and 3.

$$With\ a_i\ denoting\ for\ random\ intercept\ and\ \ b_i\ denoting\ for\ random\ slope,$$

$$the\ main\ function\ we\ fit\ is\ in\ the\ following\ form:$$

$$Phenotype_{ij} = \alpha(cov_i) + a_i + (\ \beta(cov_i) + b_i) * t_{ij} + e_{ij}\ \ for\ suject\ i\ at\ time\ j,$$

$$where\ (a_i, b_i) \sim N(0, G), e_{ij} \sim N(0, \sigma^2 R),$$

$$\alpha(cov_i)\ is\ a\ function\ of\ covariates\ described\ above,$$

$$and\ \beta(cov_i)\ is\ a\ polynomial\ function\ of\ baseline\_age_i$$

A List of Polynomial Functions (Beta(cov_i)) for each phenotype		
Phenotypic Domain	Phenotype	$\beta(cov_i)$
Body Composition	Waist	$\beta_0 + \beta_1 * BaselineAge_i + \beta_2 * BaselineAge_i^2$
Body Composition	Waist-Height Ratio	$\beta_0 + \beta_1 * BaselineAge_i + \beta_2 * BaselineAge_i^2$
Body Composition	Body Mass Index	$\beta_0 + \beta_1 * BaselineAge_i + \beta_2 * BaselineAge_i^2$
Body Composition	Lean Mass	$\beta_0 + \beta_1 * BaselineAge_i + \beta_2 * BaselineAge_i^2$
Body Composition	Appendicular Lean Mass	$\beta_0 + \beta_1 * BaselineAge_i + \beta_2 * BaselineAge_i^2$
Body Composition	Fat Mass	$\beta_0 + \beta_1 * BaselineAge_i + \beta_2 * BaselineAge_i^2$
Body Composition	Mid-Thigh Area	$\beta_0 + \beta_1 * BaselineAge_i + \beta_2 * BaselineAge_i^2$
Energetics	Resting Metabolic Rate	$\beta_0 + \beta_1 * BaselineAge_i + \beta_2 * BaselineAge_i^2$
Energetics	Peak VO2 during treadmill test	$\beta_0 + \beta_1 * BaselineAge_i$
Energetics	Peak VO2 during 400m walk	$\beta_0 + \beta_1 * BaselineAge_i$
Energetics	Cost-capacity ratio	$\beta_0 + \beta_1 * BaselineAge_i + \beta_2 * BaselineAge_i^2$
Energetics	FEV1	$\beta_0 + \beta_1 * BaselineAge_i$
Energetics	FVC	$\beta_0 + \beta_1 * BaselineAge_i$
Energetics	FEV1/FVC	$\beta_0 + \beta_1 * BaselineAge_i$
Homeostasis Mechanisms	Interleukin – 6	β_0
Homeostasis Mechanisms	C-reactive protein	β_0
Homeostasis Mechanisms	Albumin	β_0
Homeostasis Mechanisms	Hemoglobin	$\beta_0 + \beta_1 * BaselineAge_i$

Homeostasis Mechanisms	Red Blood Cell Distribution Width	β_0
Homeostasis Mechanisms	Absolute Neutrophil Count	$\beta_0 + \beta_1 * BaselineAge_i$
Homeostasis Mechanisms	Fasting Glucose	$\beta_0 + \beta_1 * BaselineAge_i$
Homeostasis Mechanisms	Systolic Blood Pressure	$\beta_0 + \beta_1 * BaselineAge_i$
Homeostasis Mechanisms	Diastolic Blood Pressure	$\beta_0 + \beta_1 * BaselineAge_i$
Homeostasis Mechanisms	Pulse Pressure	$\beta_0 + \beta_1 * BaselineAge_i + \beta_2 * BaselineAge_i^2$
Homeostasis Mechanisms	Carotid-Femoral pulse wave velocity	$\beta_0 + \beta_1 * BaselineAge_i$
Homeostasis Mechanisms	Creatinine clearance	$\beta_0 + \beta_1 * BaselineAge_i$
Homeostasis Mechanisms	Total cholesterol	$\beta_0 + \beta_1 * BaselineAge_i$
Homeostasis Mechanisms	Low-density lipoproteins	$\beta_0 + \beta_1 * BaselineAge_i$
Homeostasis Mechanisms	High-density lipoproteins	$\beta_0 + \beta_1 * BaselineAge_i + \beta_2 * BaselineAge_i^2$
Homeostasis Mechanisms	Triglyceride	$\beta_0 + \beta_1 * BaselineAge_i$
Neuroplasticity / Neurodegeneration	Total Brain Volume	$\beta_0 + \beta_1 * BaselineAge_i$
Neuroplasticity / Neurodegeneration	White Matter Volume	$\beta_0 + \beta_1 * BaselineAge_i + \beta_2 * BaselineAge_i^2$
Neuroplasticity / Neurodegeneration	Grey Matter Volume	$\beta_0 + \beta_1 * BaselineAge_i$
Neuroplasticity / Neurodegeneration	Ventricular Volume	$\beta_0 + \beta_1 * BaselineAge_i + \beta_2 * BaselineAge_i^2$
Neuroplasticity / Neurodegeneration	Fibular Nerve Conduction Velocity	$\beta_0 + \beta_1 * BaselineAge_i$
FEV1, forced expiratory volume in the first second; FVC, forced vital capacity; VO2, oxygen consumption		

Further, the difference between individual's rate of change and sex-and-age specific population's rate of change was standardized (to mean=0 and SD = 1). Those standardized values with absolute value >= 5 were considered as outliers and excluded (n excluded are small and shown in the table titled "Number of participants with repeated measurements and number of participants with rate of changes within 5SD for each phenotype" at the end of this supplemental method section). The standardized values (for each individual, for each phenotype) were then transformed to -3, -2, -1, 0, 1, 2, 3, termed "individual-phenotype-specific score", based on these standardized values (3, 2, 1 corresponding to 2.5 to 5 SD, 1.5 to 2.5 SD, 0.5 to 1.5 SD *faster/accelerated* decline in phenotypes; 0 corresponding to within 0.5 SD from changes in phenotypes; -1, -2, -3, corresponding to 0.5 to 1.5 SD, 1.5 to 2.5 SD, and 2.5 to 5 SD *slower/decelerated* decline in phenotypes). See Figure 3 for a conceptual illustration of accelerated and decelerated aging.

Step 2: For each domain, we calculated the domain-specific longitudinal phenotypic scores for each individual by averaging the available "individual-phenotype-specific score" for phenotypes within each domain, followed by quantile normalization. The main rationale for the quantile normalization in this step is due to the unequal number of phenotypes across domains. (Generally, the distribution of the domain with more phenotypes tend to appear "narrower" than the distribution of the domain with less phenotypes.)

Step 3: The global longitudinal phenotypic score was then summarized by averaging the four domain-specific longitudinal phenotypic scores, for those with all four domain-specific scores available.

Part 2: Examining the relationship between longitudinal phenotypic score(s) and functional outcomes/mortality

Part 2-1: Examining the relationship between longitudinal phenotypic score(s) and functional outcomes (physical function/cognitive function/multi-morbidity)

To estimate the relationship between global and domain-specific longitudinal phenotypic score and rate of functional decline and changes in multi-morbidities, linear mixed models with random intercept and random slope were used. Time since baseline was used as time metric, and baseline age was defined as the age at the first analytic visit. Below is the general form of linear mixed model we fit to evaluate the association between longitudinal phenotypic score and rate of changes in physical and cognitive functions:

$$With\ a_i\ denoting\ for\ random\ intercept\ and\ \ b_i\ denoting\ for\ random\ slope,$$

$$the\ main\ function\ we\ fit\ is\ in\ the\ following\ form:$$

$$Function_{ij} = \alpha(cov_i) + a_i + (\ \beta(cov_i) + b_i) * t_{ij} + e_{ij}\ \ for\ suject\ i\ at\ time\ j,$$

$$where\ (a_i, b_i) \sim\ N(0, G), e_{ij} \sim N(0, \sigma^2 R),$$

$$\alpha(cov_i)\ is\ a\ function\ of\ covariates\ (ex{:}\ baseline\ age_i\ , sex_i\ ),$$

$$and\ \beta(cov_i)\ is\ a\ function\ of\ longitudinal\_phenotypic\_score_i\ , baseline\_age_i\ , and\ sex_i$$

Specifically, for cognitive function, the models included sex, baseline age, years of education, race, longitudinal phenotypic aging score (global longitudinal phenotypic score or domain-specific longitudinal phenotypic scores), time since baseline age, interaction between baseline age and time since baseline age, interaction between sex and time since baseline age, and interaction between longitudinal phenotypic aging score and time since baseline age. The data used for modeling the rate of changes in cognitions come from all the cognitive tests given at age 50 and above.

For physical function, the models included sex, quadratic function of baseline age, height, weight, longitudinal phenotypic aging score, time since baseline age, interaction between baseline age and time since baseline age, interaction between sex and time since baseline age, and interaction between longitudinal phenotypic aging score and time since baseline age.

For multi-morbidity index, the model included sex, quadratic function of baseline age, longitudinal phenotypic aging score, time since baseline age, interaction between baseline age and time since baseline age, interaction between sex and time since baseline age, and interaction between the longitudinal phenotypic aging score and time since baseline age.

With these linear mixed models, the coefficient for the interaction term between longitudinal phenotypic aging score and time since baseline age can be interpreted as the difference in rate of change per one point higher in longitudinal phenotypic aging score (i.e. the accelerated aging) [for global longitudinal phenotypic score or domain-specific longitudinal phenotypic scores]. Similarly, the coefficient for the interaction term between baseline age and time since baseline age can be interpreted as the difference in rate of change per one year older in chronological age. Because the scales of cognitive functions, physical functions, and multi-morbidities are different, to improve the interpretability of results, we translated the results as "age-equivalent," which can be interpreted as the equivalent effect of the number of years chronological age increase on rate of changes per one point higher in summarized score (global longitudinal phenotypic score or domain-specific longitudinal phenotypic scores).

To visualize the results, we plotted the scatterplot between summarized global score and slopes of changes in cognitive functions, physical functions, and multi-morbidities. To facilitate the understanding of our results, the rate of changes in physical and cognitive functions and multi-morbidities were reported in the Supplemental Table 3, and the incremental changes in rate of changes due to older chronological age, estimated by the coefficients interaction term between longitudinal phenotypic aging score and time since baseline age, were reported in the Supplemental Table 4.

To better understand the rank contribution of different domain-specific longitudinal phenotypic scores to the changes in physical and cognitive functions, we first regressed the rate of changes on each domain-specific longitudinal phenotypic score separately, and then ranked them by the amount of variability explained using adjusted r-squared.

Age-stratified scatterplots were also provided to explore the relationship between the global longitudinal phenotypic score and changes in physical and cognitive functions across different age strata. For physical functions, cutoffs for three groups are 50 and 80. Because for cognitive functions some measures were obtained only in participants who were age 50 and above at the time of their visit, cutoff for three groups are 65 and 80. Since the relationship between longitudinal phenotypic score and changes in physical/cognitive functions appear to be stronger

among older adults, we further tested whether the relationship between global longitudinal phenotypic score and change in physical and cognitive functions differed by age. To do this, we included all the two-way and three-way interactions between baseline age, time since baseline age, and the global longitudinal phenotypic score, so that the three-way interaction term empirically tests whether the relationship between global longitudinal phenotypic score and change in physical and cognitive functions is stronger among older participants.

Linear mixed effects models were performed using R package `lme4` (version 1.1.23). Part of data wrangling were performed using R package `dplyr` (version 0.8.5), and `tidyverse` (version 1.3.0).

Part 2-2: Examining the relationship between global longitudinal phenotypic score and mortality

To understand the relationship between the global longitudinal phenotypic score and mortality, we used survival analysis with both semi-parametric Cox models and parametric Weibull distribution to quantify the relationship between summarized global score and mortality risk using age starting from 60 years old as timescale with adjustment for age, sex and education. For the Cox model, we found no evidence of violation of the proportional hazard assumption in the analysis concerning the global longitudinal phenotypic score. For the parametric survival analysis, we fitted the survival curve with Weibull distribution. We further reported time ratios, which is better for clinical interpretation and physician-patient communication. These analyses were conducted using Stata version 15.1 (StataCorp, College Station, Texas), R packages `flexsurv` (version 1.1.1) and `survival` (version 3.2.7).

Part 3: Evaluation the association between cross-sectional measurements and changes in physical and cognitive functions

To understand the potential difference between the cross-sectional aging summary and our global longitudinal phenotypic scores mortality, we also computed the association between global cross-sectional phenotypic score, 6 epigenetic age acceleration measurements, and changes in physical and cognitive functions.

The global cross-sectional phenotypic score is created with the same approach as the global longitudinal phenotypic score except that only cross-sectional data are used. Specifically, in the first step, we calculated the difference between cross-sectional phenotypic measurement and the age-and-sex specific population average (which is created from cross-sectional data and fitted with the best fit polynomial curve to capture non-linearity). The second and the third step replicate those used to calculate the global longitudinal phenotypic score.

As to measurement of epigenetic age accelerations, we included 6 popular epigenetic age accelerations (Horvath's clock – both intrinsic and epigenetic age acceleration, Hannum's clock, Levine's PhenoAge, Lu's GrimAge, and Belsky's PaceOfAging Estimation) (2-7). In BLSA, DNA methylation was assayed using DNA extracted from blood samples collected at visits between November 1993 and March 2010 (8). CpG methylation status of 485,577 CpG sites was

determined using the Illumina Infinium HumanMethylation450 BeadChip (Illumina Inc., San Diego, CA) per the manufacturer's protocol. Data processing included NOOB and BMIQ normalization using R package "minfi" (9). Multi-dimension scaling-defined outliers, as well as sex and SNP discordant samples were excluded in quality control. Epigenetic ages were calculated using the Horvath online calculator (https://dnamage.genetics.ucla.edu/) or "projector" package (https://github.com/danbelsky/DunedinPoAm38). The following chronological age independent epigenetic measures were then derived: intrinsic and epigenetic age acceleration—"*IEAA*" & "*EEAA*"(10), Hannum age acceleration—"*AgeAccelerationResidualHannum*", PhenoAge acceleration—"AgeAccelPheno", GrimAge acceleration—"*AgeAccelGrim*", and methylation-based pace of aging estimation — " *Dunedin_PoAm_38*".

To estimate the relationship between aging summaries derived from cross-sectional data (global cross-sectional phenotypic score and epigenetic age acceleration measurements) and rate of functional decline and changes in multi-morbidities, we used linear mixed models with random intercepts and random slopes. This is the same approach as what described in "**Part 2-1: Examining the relationship between longitudinal phenotypic score(s) and functional outcomes (physical function/cognitive function/multi-morbidity)",** except that the longitudinal phenotypic scores are replaced by these aging summaries based on cross-sectional data.

All the analysis was performed using R 3.6.2, Stata version 15.1 (StataCorp, College Station, Texas), and SAS 9.4. The point estimates and 95% confidence intervals of the associations are reported. Two-sided tests were used, and the displayed p-value was not adjusted for multiple comparisons.

Number of participants with repeated measurements and number of participants with rate of changes within 5SD for each phenotype		
Aging Phenotype	Number of participants with repeated measurements	Number of participants with repeated measurements (and the slope is ***within*** 5 SD)
	N	N
Body composition domain		
Waist (cm)	1212	1210
Waist-height ratio	1212	1210
Body mass index (kg/m^2)	1256	1252
Lean mass (kg)	1117	1116
Appendicular lean mass (kg)	1117	1113
Fat mass (kg)	1117	1116
Mid-thigh area (mm^2)	987	984
Energetics Domain		
Resting metabolic rate (kcal/day)	926	926
Peak VO_2 (400m walk) (ml/kg/min)	836	835
Peak VO_2 (treadmill) (ml/kg/min)	836	832
Cost-capacity ratio	755	755
FEV_1 (L)	955	952
FVC (L)	955	952
FEV_1/FVC	955	949
Homeostasis Domain		
Interleukin - 6 (pg/mL)	1277	1277
C-Reactive Protein (mg/L)	1134	1134
Albumin (g/dL)	1164	1163
Hemoglobin (g/dL)	1201	1199

Red blood cell distribution width (%)	1179	1177
Absolute neutrophil count (cells/uL)	1200	1198
Fasting glucose (mg/dL)	1084	1084
Systolic blood pressure (mmHg)	1013	1012
Diastolic blood pressure (mmHg)	1013	1009
Pulse pressure (mmHg)	1013	1011
Carotid-Femoral pulse wave velocity (m/s)	1022	1020
Creatinine clearance (ml/min/1.73*m^2)	1074	1074
Total cholesterol (mg/dL)	1156	1156
Low-density lipoproteins (mg/dL)	1156	1156
High-density lipoproteins (mg/dL)	1155	1155
Triglyceride (mg/dL)	1163	1163
Neurodegeneration/Neuroplasticity domain		
Total brain volume (cm^3)	572	572
White matter (cm^3)	572	570
Grey matter (cm^3)	572	571
Ventricular volume (cm^3)	572	572
Fibular nerve conduction velocity (m/s)	966	965

FEV1, forced expiratory volume in the first second; FVC, forced vital capacity; VO2, oxygen consumption

Reference for Supplemental Method III - Statistical Analysis

1. Muller C, Schillert A, Rothemeier C, Tregouet DA, Proust C, Binder H, et al. Removing Batch Effects from Longitudinal Gene Expression - Quantile Normalization Plus ComBat as Best Approach for Microarray Transcriptome Data. PLoS One. 2016;11(6):e0156594.
2. Belsky DW, Caspi A, Arseneault L, Baccarelli A, Corcoran DL, Gao X, et al. Quantification of the pace of biological aging in humans through a blood test, the DunedinPoAm DNA methylation algorithm. Elife. 2020;9.
3. Lu AT, Quach A, Wilson JG, Reiner AP, Aviv A, Raj K, et al. DNA methylation GrimAge strongly predicts lifespan and healthspan. Aging (Albany NY). 2019;11(2):303-27.
4. Levine ME, Lu AT, Quach A, Chen BH, Assimes TL, Bandinelli S, et al. An epigenetic biomarker of aging for lifespan and healthspan. Aging (Albany NY). 2018;10(4):573-91.
5. Horvath S. DNA methylation age of human tissues and cell types. Genome Biol. 2013;14(10):R115.
6. Hannum G, Guinney J, Zhao L, Zhang L, Hughes G, Sadda S, et al. Genome-wide methylation profiles reveal quantitative views of human aging rates. Mol Cell. 2013;49(2):359-67.
7. Bocklandt S, Lin W, Sehl ME, Sanchez FJ, Sinsheimer JS, Horvath S, et al. Epigenetic predictor of age. PLoS One. 2011;6(6):e14821.
8. Kuo PL, Moore AZ, Lin FR, Ferrucci L. Epigenetic Age Acceleration and Hearing: Observations From the Baltimore Longitudinal Study of Aging. Front Aging Neurosci. 2021;13:790926.
9. Aryee MJ, Jaffe AE, Corrada-Bravo H, Ladd-Acosta C, Feinberg AP, Hansen KD, et al. Minfi: a flexible and comprehensive Bioconductor package for the analysis of Infinium DNA methylation microarrays. Bioinformatics. 2014;30(10):1363-9.
10. Horvath S, Gurven M, Levine ME, Trumble BC, Kaplan H, Allayee H, et al. An epigenetic clock analysis of race/ethnicity, sex, and coronary heart disease. Genome Biol. 2016;17(1):171.

Supplemental Figure 1. Follow-up structure of the 968 participants with global longitudinal phenotypic score

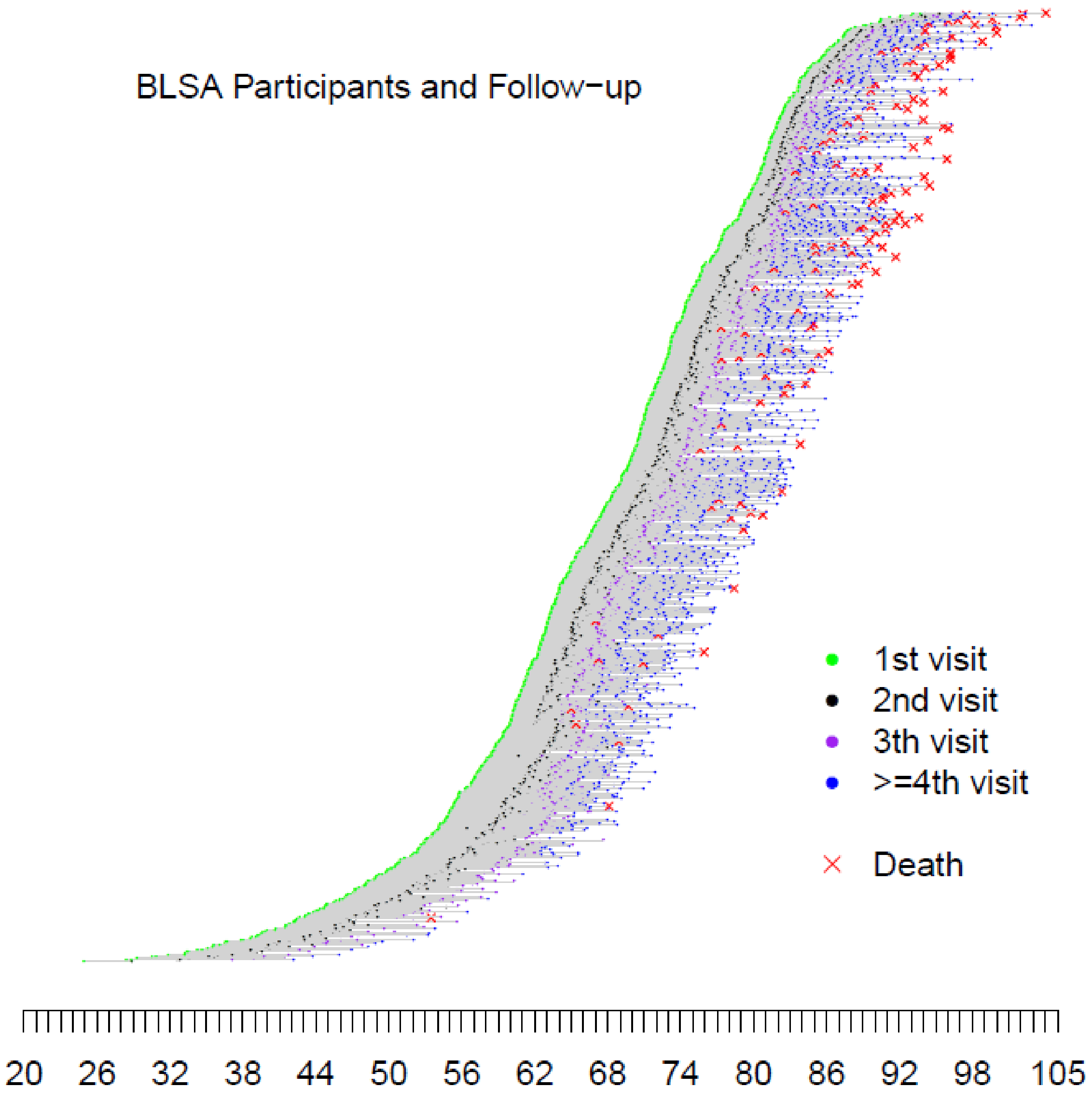

Supplemental Figure 2. Rate of changes in 35 aging phenotypes among male participants

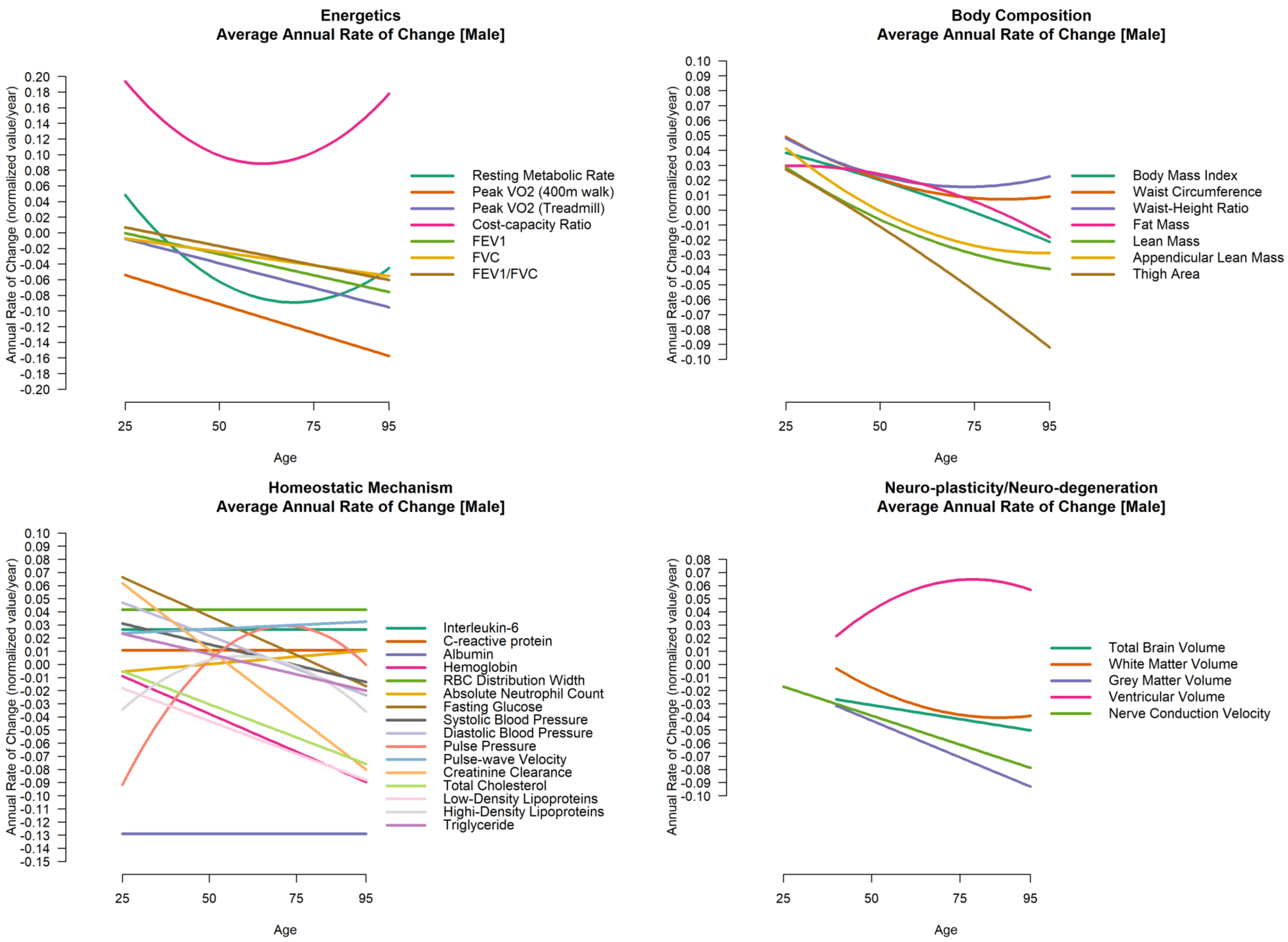

Abbreviation: VO_2 = oxygen uptake, FVC = forced vital capacity, FEV_1 = forced expiratory volume in the first second, RBC = red blood cell

Supplemental Figure 3. Rate of changes in 35 aging phenotypes among female participants

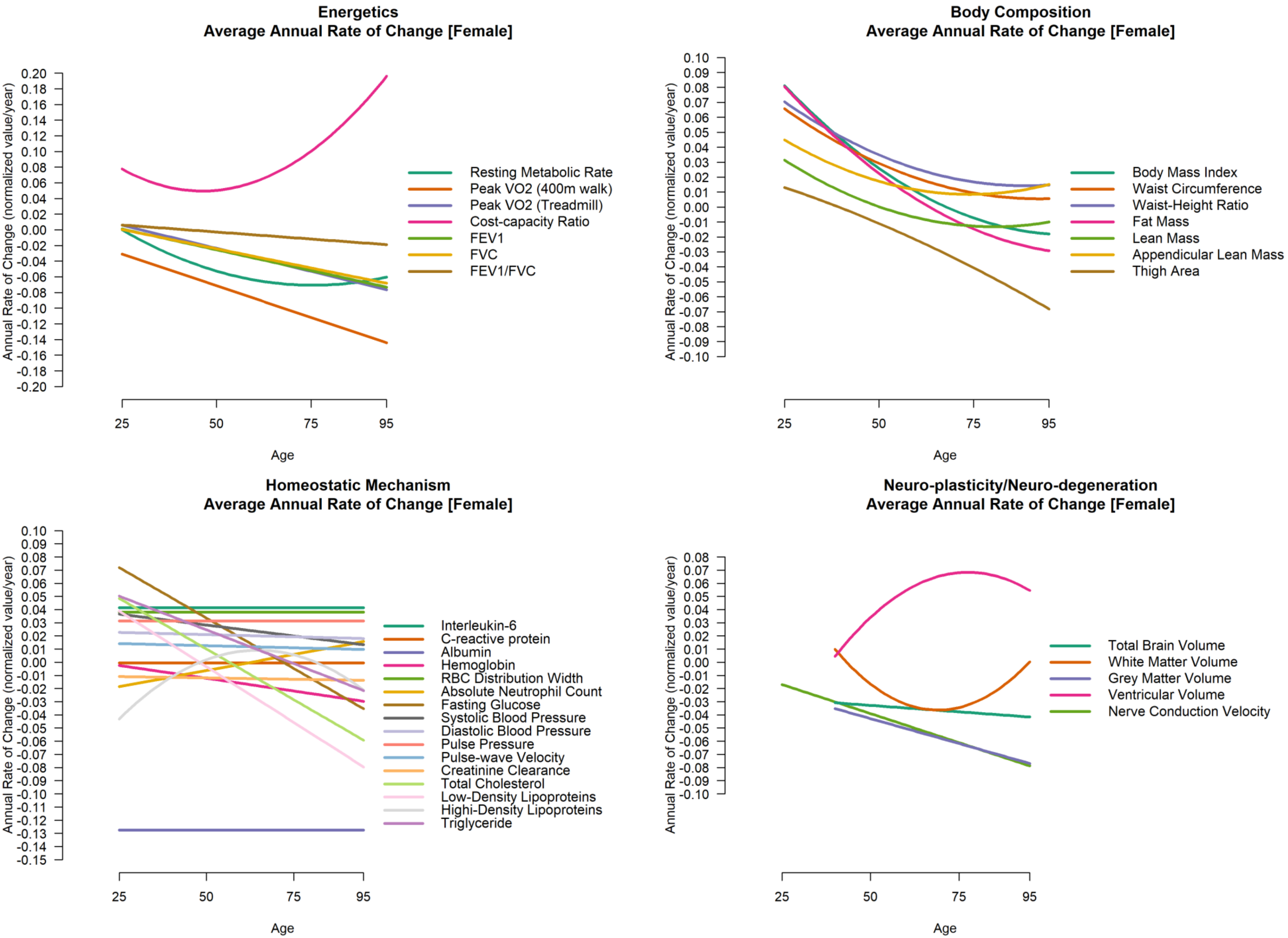

Abbreviation: VO_2 = oxygen uptake, FVC = forced vital capacity, FEV_1 = forced expiratory volume in the first second, RBC = red blood cell

Supplemental Figure 4. Domain-specific Longitudinal Phenotypic Scores

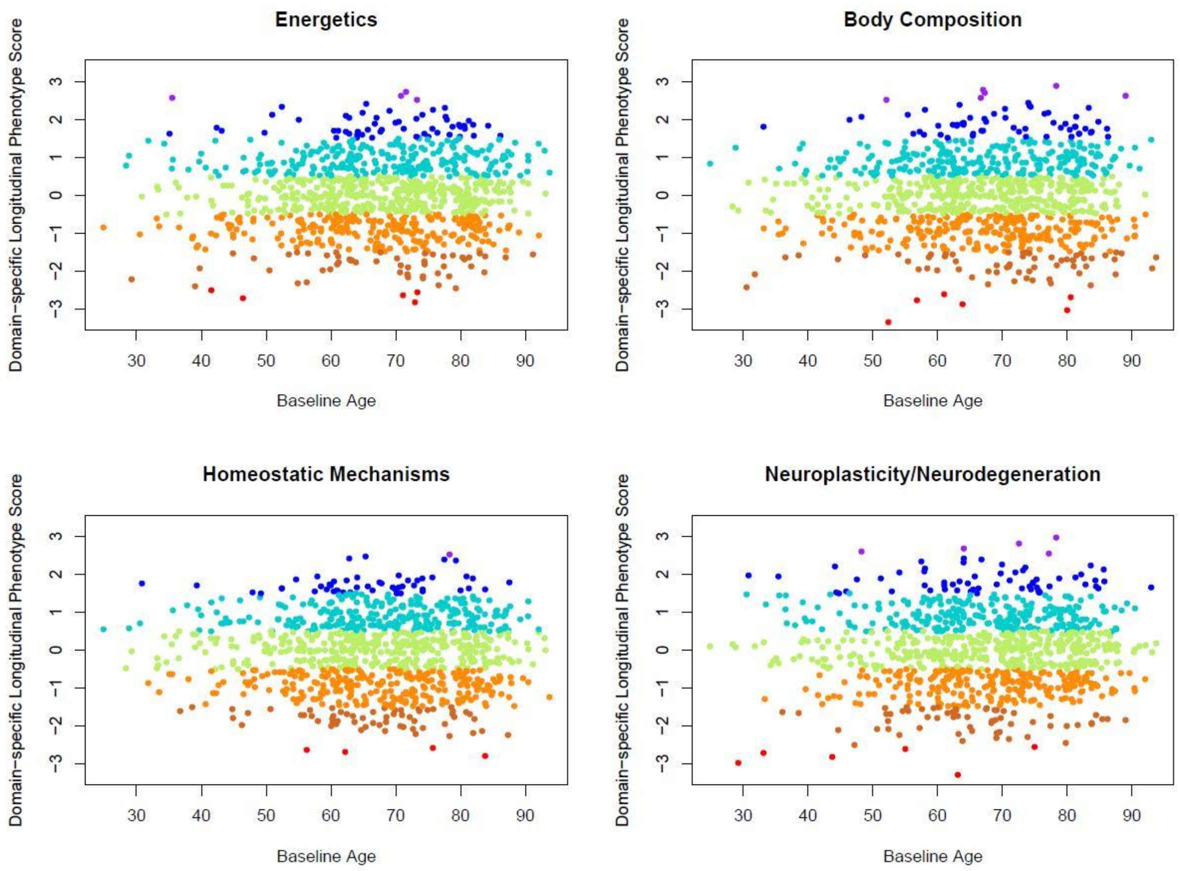

The above figures show the distribution of domain-specific scores over baseline age. The colors are shown based on the values ("red" for >2.5, "pink" for 1.5 ~ 2.5, “orange” for 0.5 ~ 1.5, “green” for -0.5 ~ 0.5, “cyan” for -1.5 ~ -0.5, “blue” for -2.5 ~ -1.5, “purple” for < -2.5).

Supplemental Figure 5. Global Longitudinal Phenotypic Score

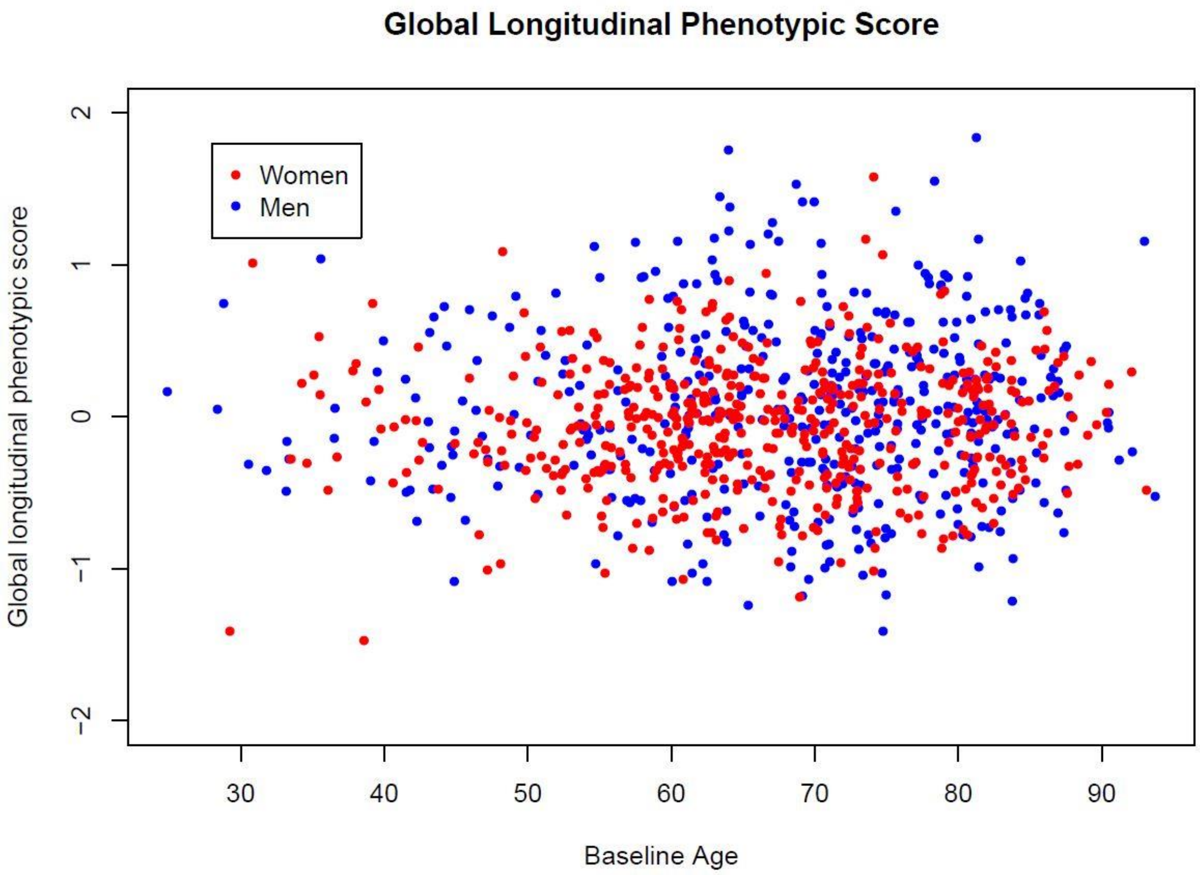

The above figures show the distribution of global longitudinal phenotypic scores over baseline age. Red dots are for women, and blue dots are for men.

Supplemental Figure 6. Age Equivalence of One-Point Difference in Domain-specific Longitudinal Phenotypic Scores

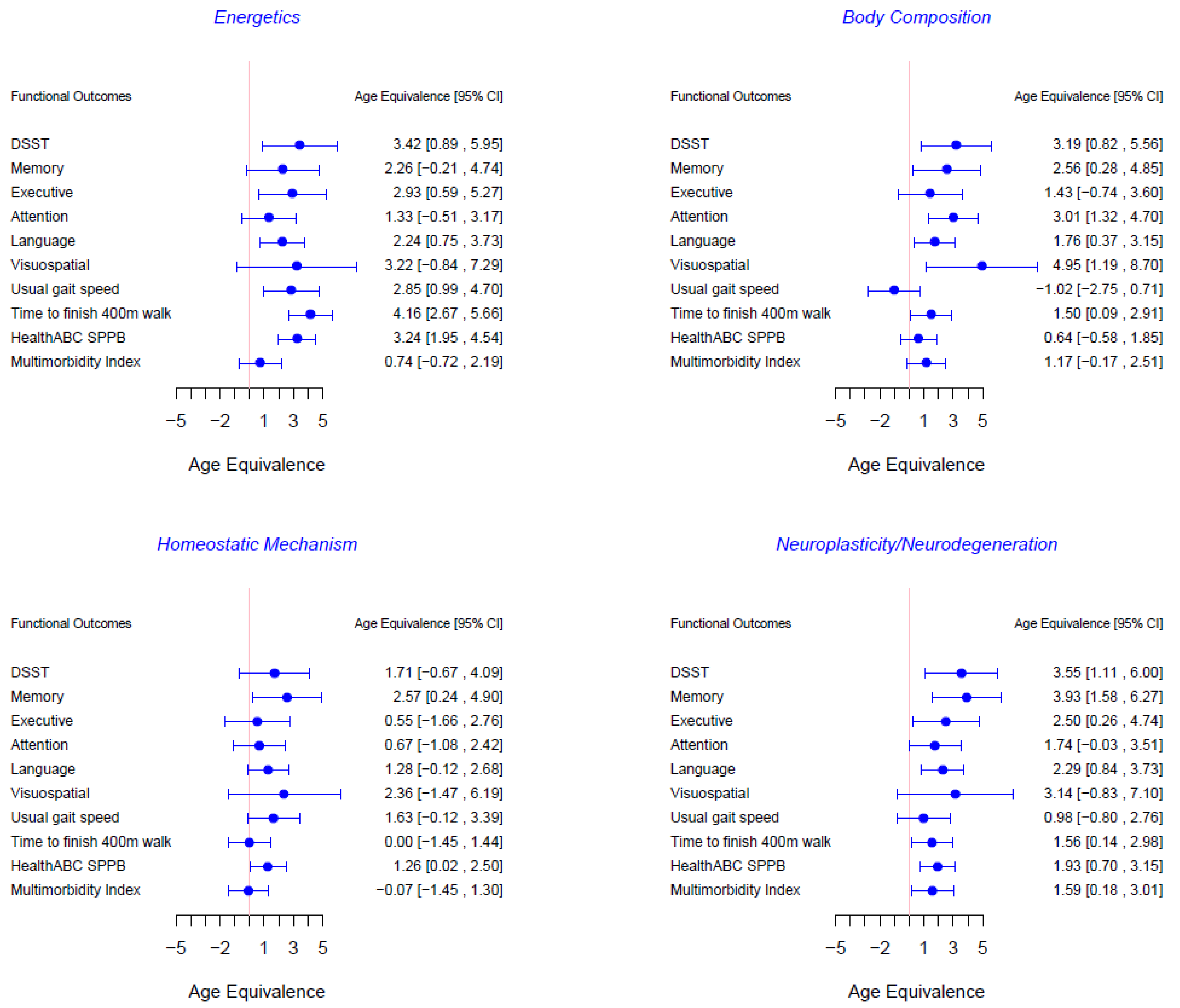

The above figures show the age equivalence of domain-specific longitudinal phenotypic scores. Age equivalence presented here is a scaled regression coefficient between the domain-specific longitudinal phenotypic score and rate of changes in functions, meaning to how many years older in chronological age is a point higher in the domain-specific longitudinal phenotypic score equivalent. Results are shown as point estimates with 95% confidence interval. [Number of participants: n = 921 (DSST), n = 922 (memory), n = 929 (executive function), n = 929 (attention), n = 929 (language), n = 919 (visual spatial ability), n = 968 (usual gait speed), n = 943 (time to finish 400m walk), n = 968 (HealthABC SPPB), n = 828 (multi-morbidity index), also see Supplemental Table 1B for more details".]

Abbreviation: HealthABC = Health Aging, and Body Composition; SPPB = short physical performance battery

Supplemental Figure 7. Predicted probability of death by global longitudinal phenotypic score from 60 years old.

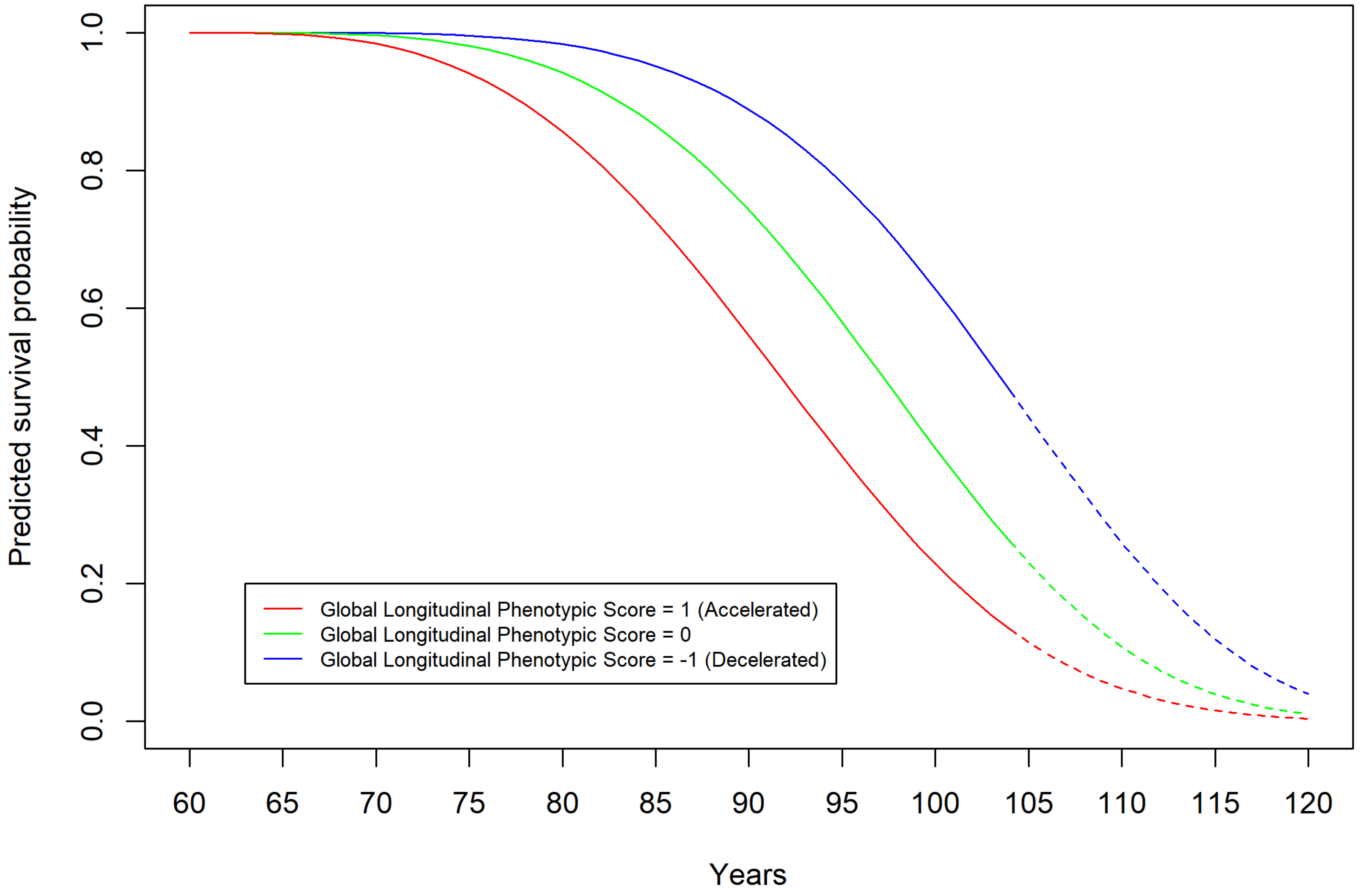

The survival curves are derived from the parametric survival models we fit for the population aged 60 and above. Because the oldest observed death is at age 104, the extrapolated part (above 104 years) is shown in dashed lines.

Supplemental Figure 8. Age-stratified scatterplots for the relationship between global longitudinal phenotypic score and changes in physical functions.

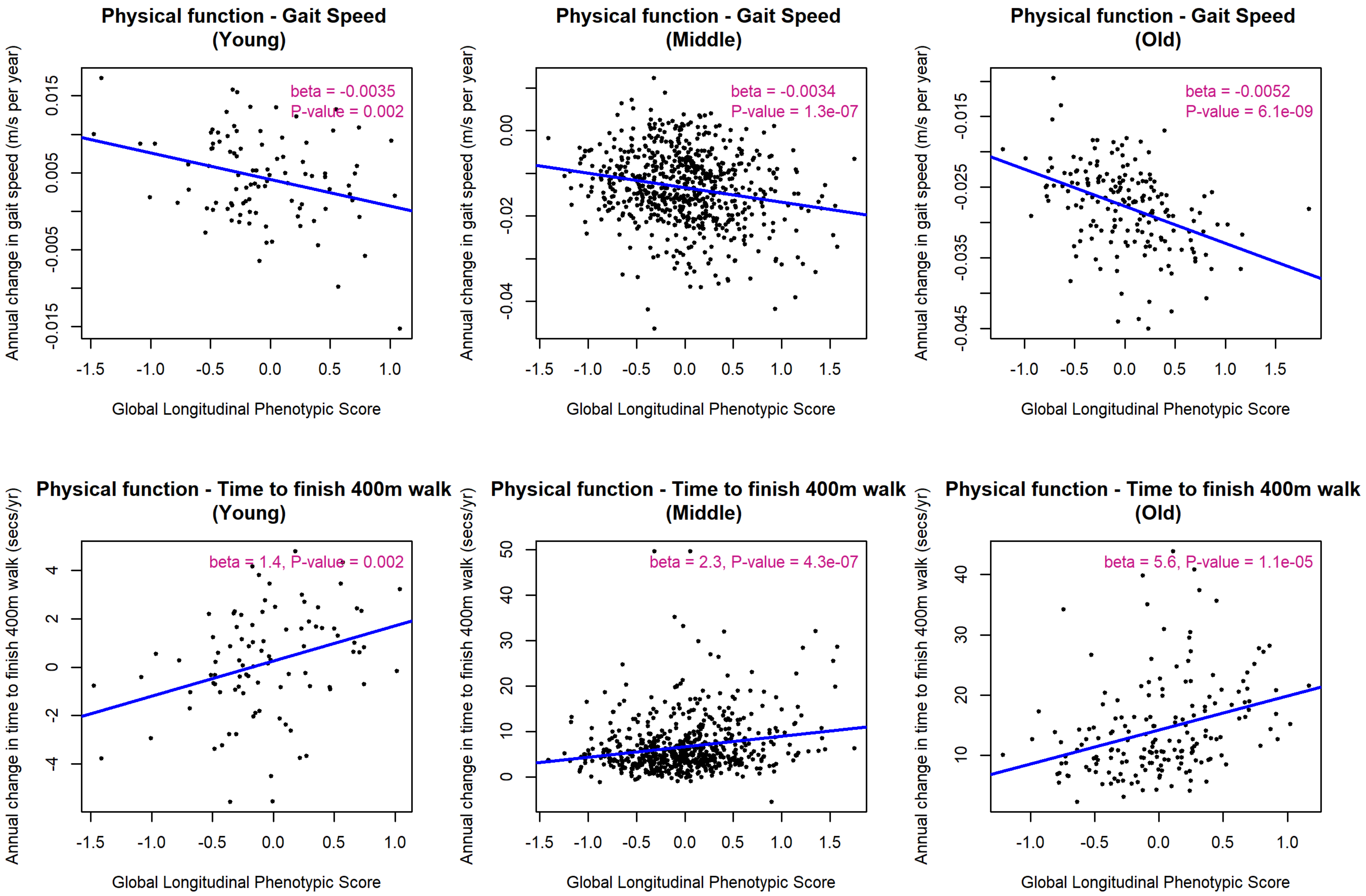

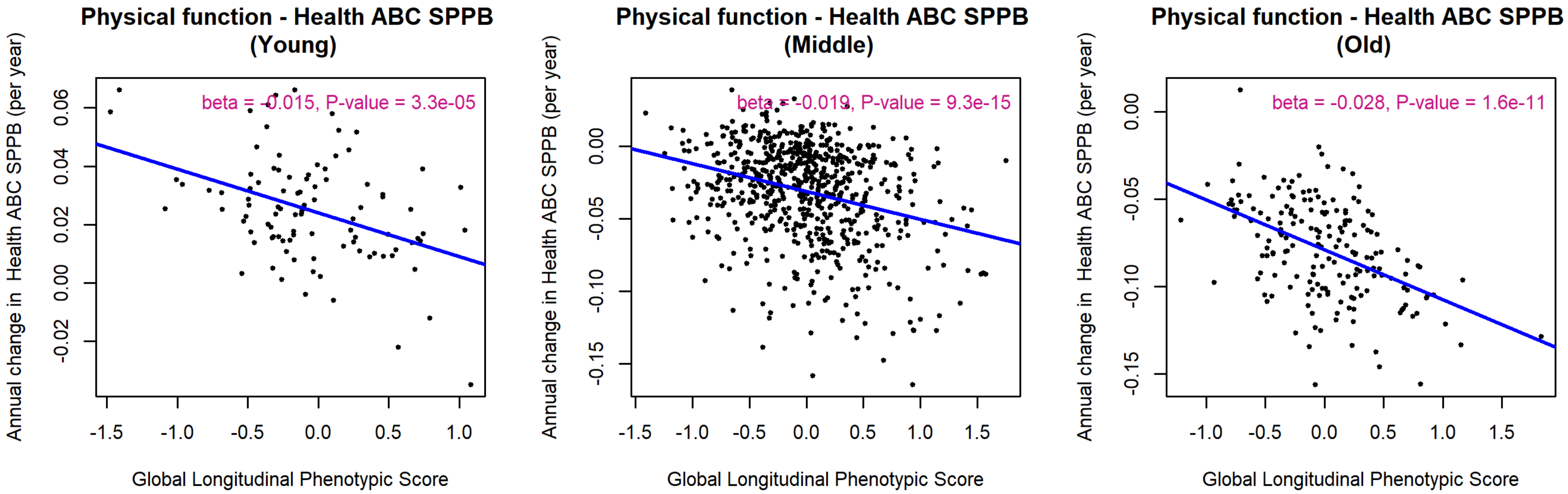

Higher global longitudinal phenotypic score indicates accelerated phenotypic aging trajectories. Higher annual decrease in gait speed and Health hABC SPPB scores, along with higher annual increase in the time to 400m walk indicate faster decline in physical function. The three-age groups are defined as following: young (<=50 years), middle (51-79 years), old (80+ years). To formally test the hypothesis that the relationship between global longitudinal phenotypic score and changes in physical functions increase with baseline age we tested three-way interactions, and they were all significant ($p = 0.002$ for usual gait speed, and $p < 0.001$ for both time to finish 400 m walk and HealthABC SPPB). Two-sided tests were used, and the displayed p-value was not adjusted for multiple comparisons.

Abbreviation: Health ABC = Health Aging, and Body Composition; SPPB = short physical performance battery

Supplemental Figure 9. Age-stratified scatterplots for the relationship between global longitudinal phenotypic score and changes in cognitive functions

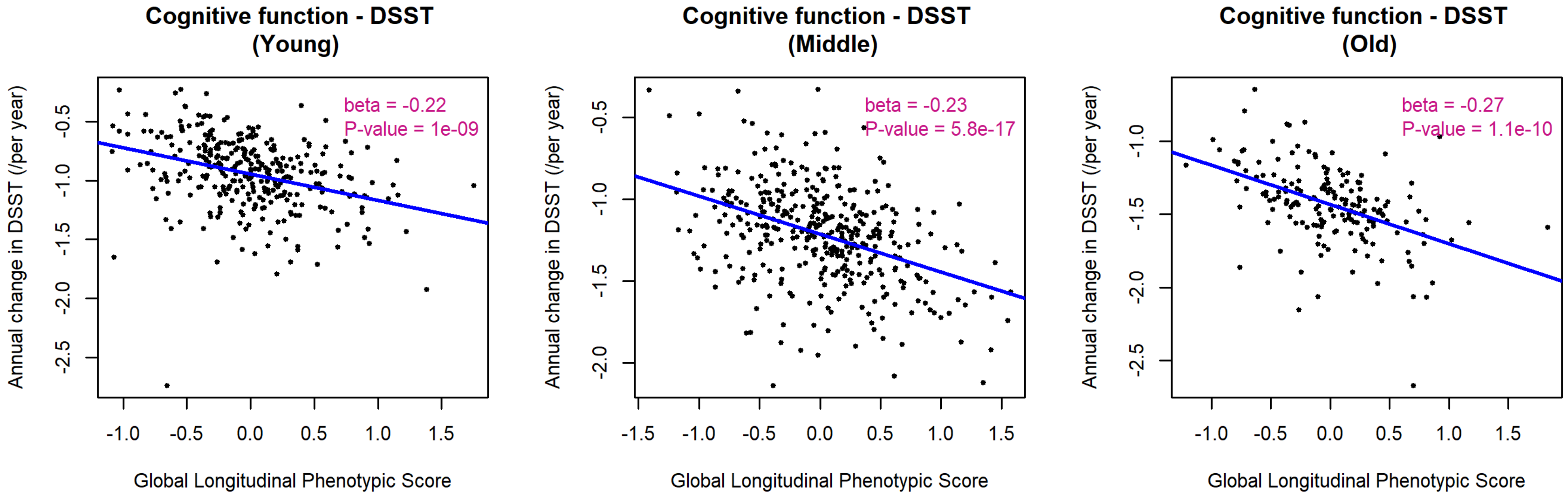

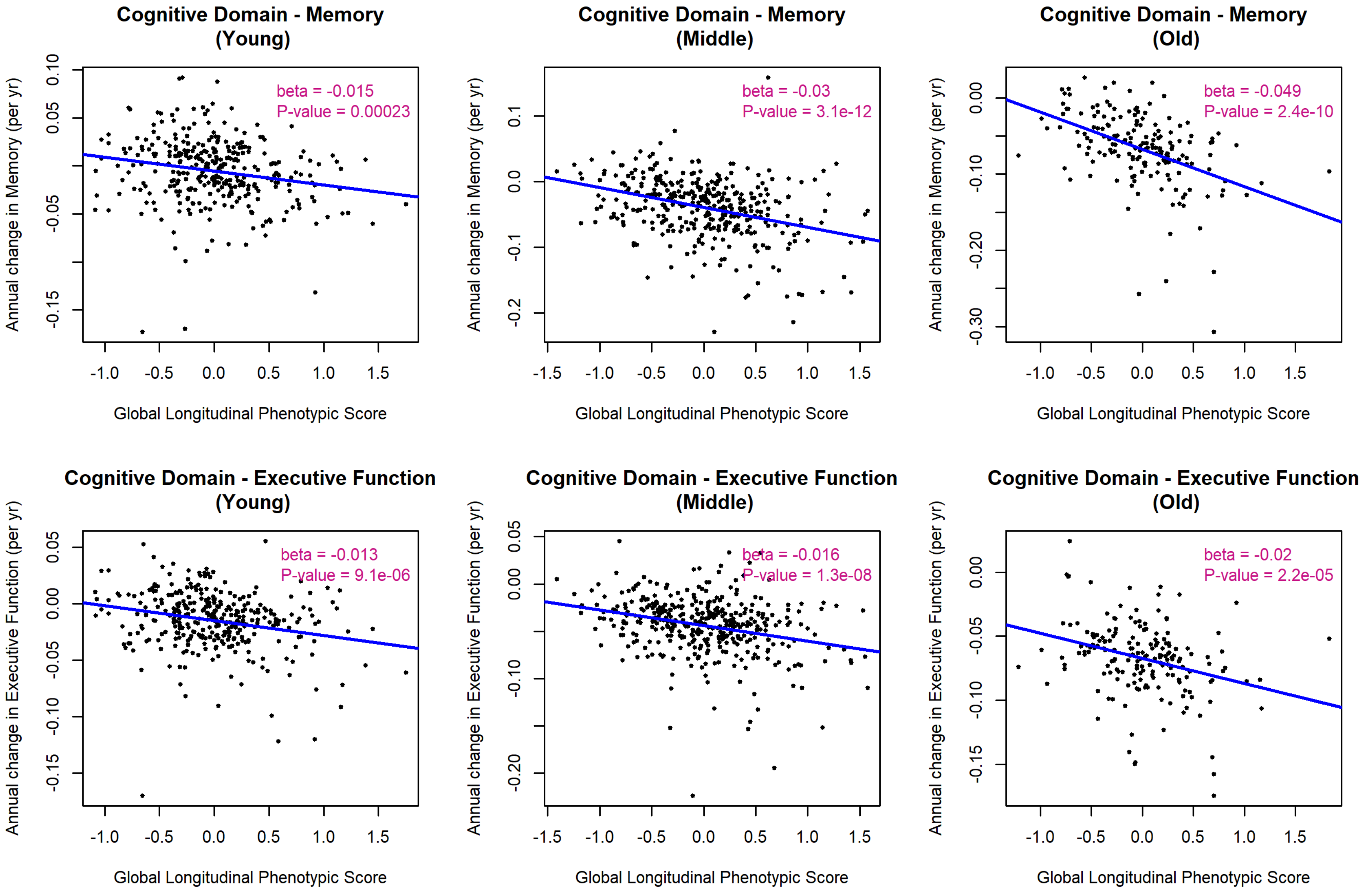
Cognitive Domain - Memory (Young)
beta = -0.015
P-value = 0.00023
Cognitive Domain - Memory (Middle)
beta = -0.03
P-value = 3.1e-12
Cognitive Domain - Memory (Old)
beta = -0.049
P-value = 2.4e-10
Annual change in Memory (per yr)
Global Longitudinal Phenotypic Score
Cognitive Domain - Executive Function (Young)
beta = -0.013
P-value = 9.1e-06
Cognitive Domain - Executive Function (Middle)
beta = -0.016
P-value = 1.3e-08
Cognitive Domain - Executive Function (Old)
beta = -0.02
P-value = 2.2e-05
Annual change in Executive Function (per yr)
Global Longitudinal Phenotypic Score

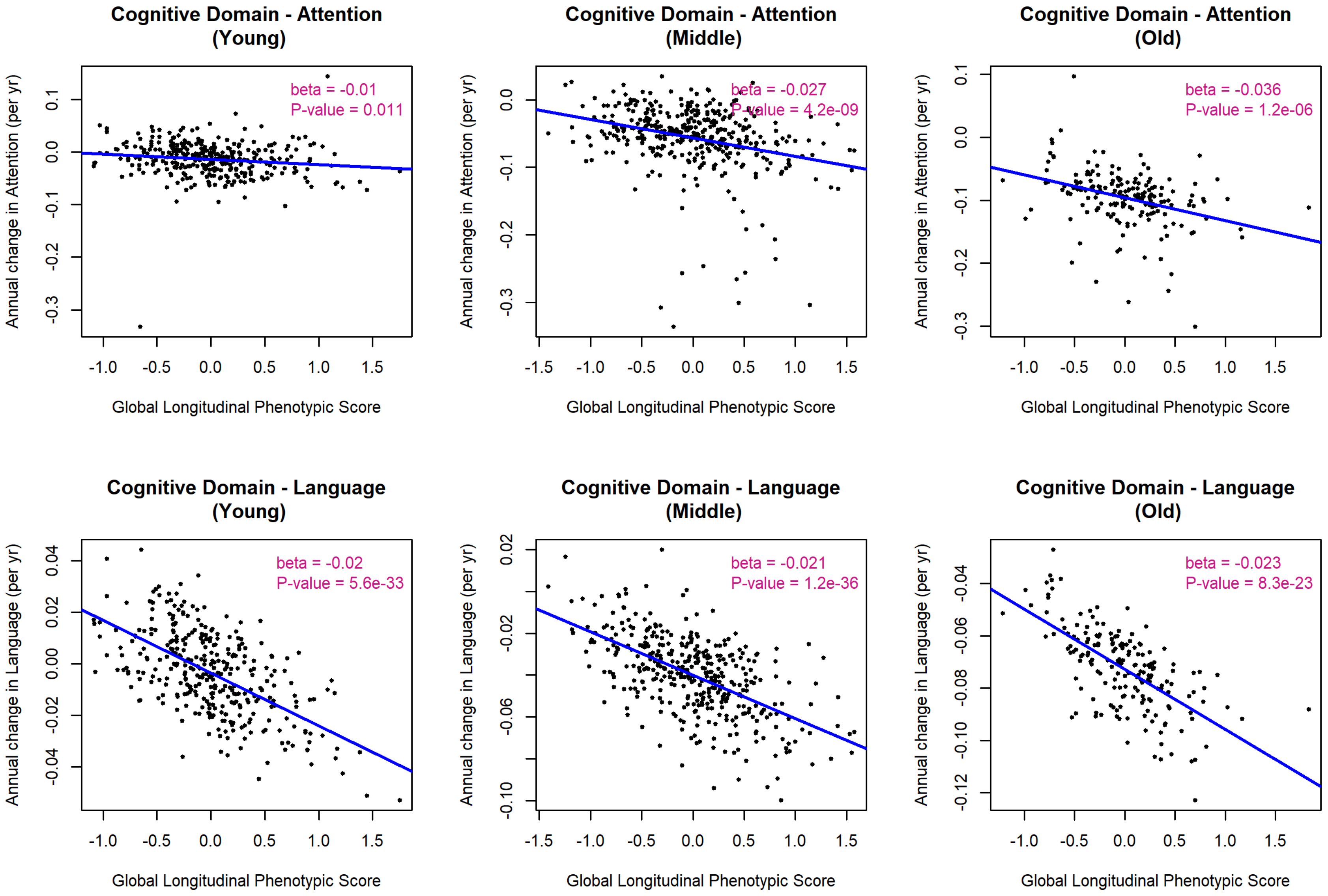

Cognitive Domain - Attention (Young)
beta = -0.01
P-value = 0.011
Cognitive Domain - Attention (Middle)
beta = -0.027
P-value = 4.2e-09
Cognitive Domain - Attention (Old)
beta = -0.036
P-value = 1.2e-06
Annual change in Attention (per yr)
Cognitive Domain - Language (Young)
beta = -0.02
P-value = 5.6e-33
Cognitive Domain - Language (Middle)
beta = -0.021
P-value = 1.2e-36
Cognitive Domain - Language (Old)
beta = -0.023
P-value = 8.3e-23
Annual change in Language (per yr)
Global Longitudinal Phenotypic Score

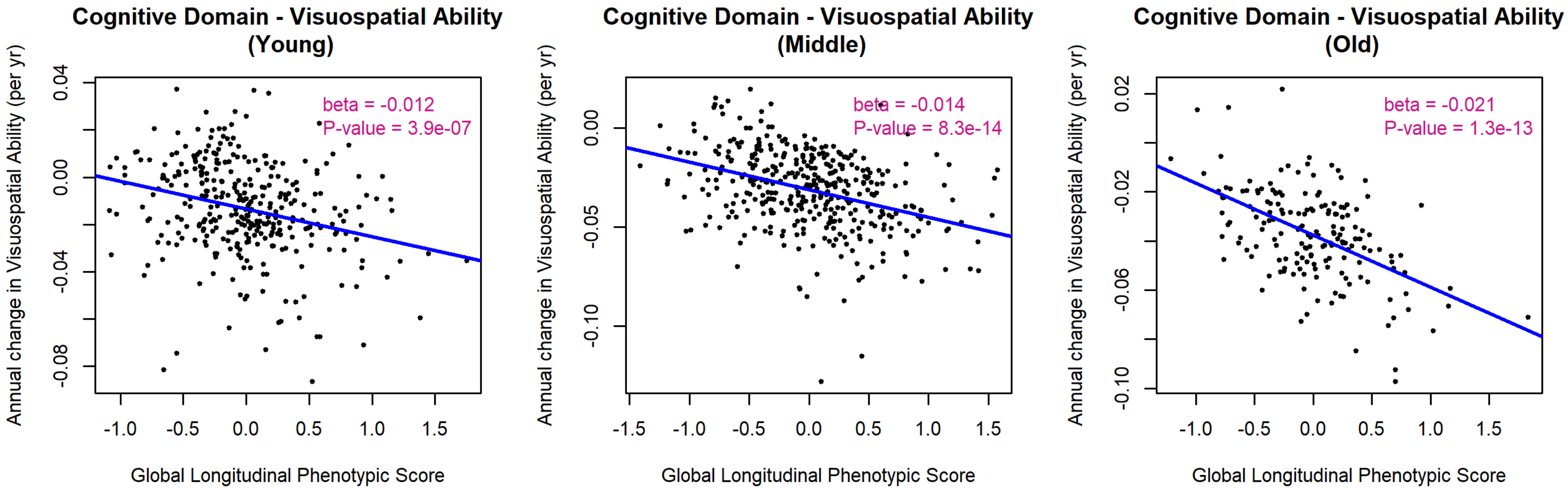

Higher global longitudinal phenotypic score indicates accelerated phenotypic aging trajectories. Higher annual decrease in Digital Symbol Substitution Test (DSST), and executive function, attention, memory, language, and visuospatial ability indicate faster decline in cognitive function. Memory score is constructed by the average of standardized immediate recall and long-delay free recall from California Verbal Learning. Language score is calculated as the average of standardized letter fluency and standardized category fluency. Attention score is calculated as the average of standardized log-transformed Trail Making Tests Part A and Digit Span Forward. Executive function is calculated as the average of the standardized log-transformed Trail Making Tests Part B and Digit Span Backward. Visuospatial ability is estimated by the standardized Cart Rotations test. The three-age groups are defined as following: young (50-65 years), middle (66-79 years), old (80+ years). To formally test the hypothesis that whether the relationship between global longitudinal phenotypic score and changes in cognitive functions increase with baseline age we tested the three-way interactions. The three-way interactions were significant for memory ($p < 0.001$) and attention ($p = 0.001$), but not significant for DSST ($p = 0.472$), executive function ($p = 0.124$), language ($p = 0.162$), and visuospatial ability ($p=0.051$). Two-sided tests were used, and the displayed p-value was not adjusted for multiple comparisons.

Supplemental Figure 10. Age Equivalence of Epigenetic Age Acceleration

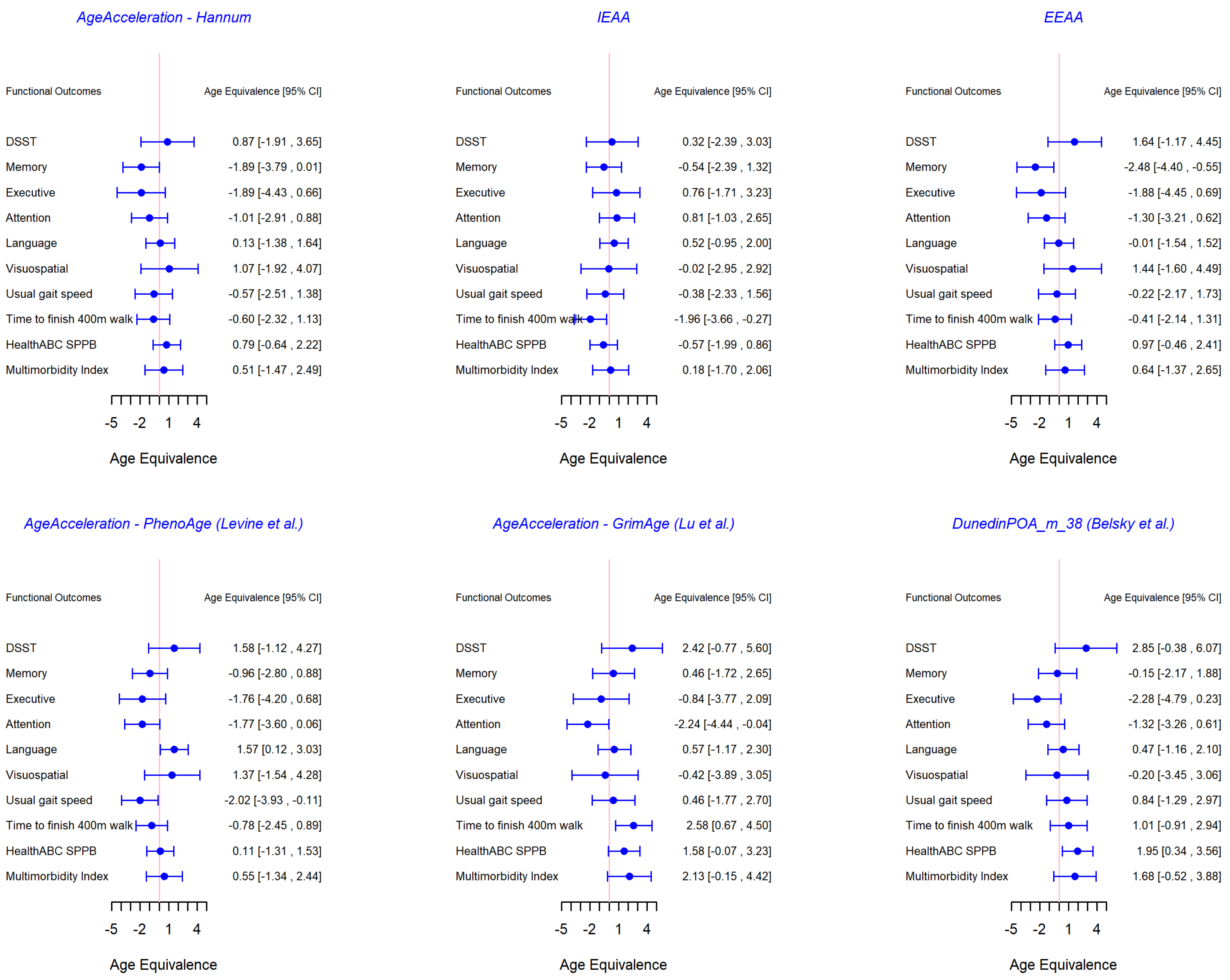

The plot shows the estimated age-equivalence of one standard deviation difference in epigenetic age acceleration for different functional outcomes, including cognitive function, physical function, and multi-morbidities. Epigenetic age clocks considered include Hannum's clock, Horvath's clock, Levine's methylation-based estimation of PhenoAge, Lu's GrimAge, and Belsky's methylation-based pace of aging estimator. Except for Belsky's DunedinPOA_m_38, the epigenetic age accelerations were calculated based on the instruction on Horvath's website (http://dnamage.genetics.ucla.edu/). Belsky's pace of ageing was not designed to use "years" as unit and thus scaled at a unit of 1 standard deviation within analytic samples. Age equivalence presented here is a scaled regression coefficient between the epigenetic age acceleration and rate of changes in functions, meaning to how many years older in chronological age is one standard deviation higher in epigenetic age acceleration equivalent. Results are shown as point estimates with 95% confidence interval. [Number of participants: n = 489 (DSST), n = 487 (memory), n = 491 (executive function), n = 491 (attention), n = 491 (language), n = 485 (visual spatial ability), n = 504 (usual gait speed), n = 487 (time to finish 400m walk), n = 504 (HealthABC SPPB), n = 447 (multi-morbidity index)] [Abbreviation: IEAA = intrinsic epigenetic age acceleration, EEAA = extrinsic epigenetic age acceleration, HealthABC = Health Aging, and Body Composition, SPPB = short physical performance battery]

Supplemental Figure 11. Pairwise Cross-sectional Correlation between the 35 phenotypes considered in our analysis

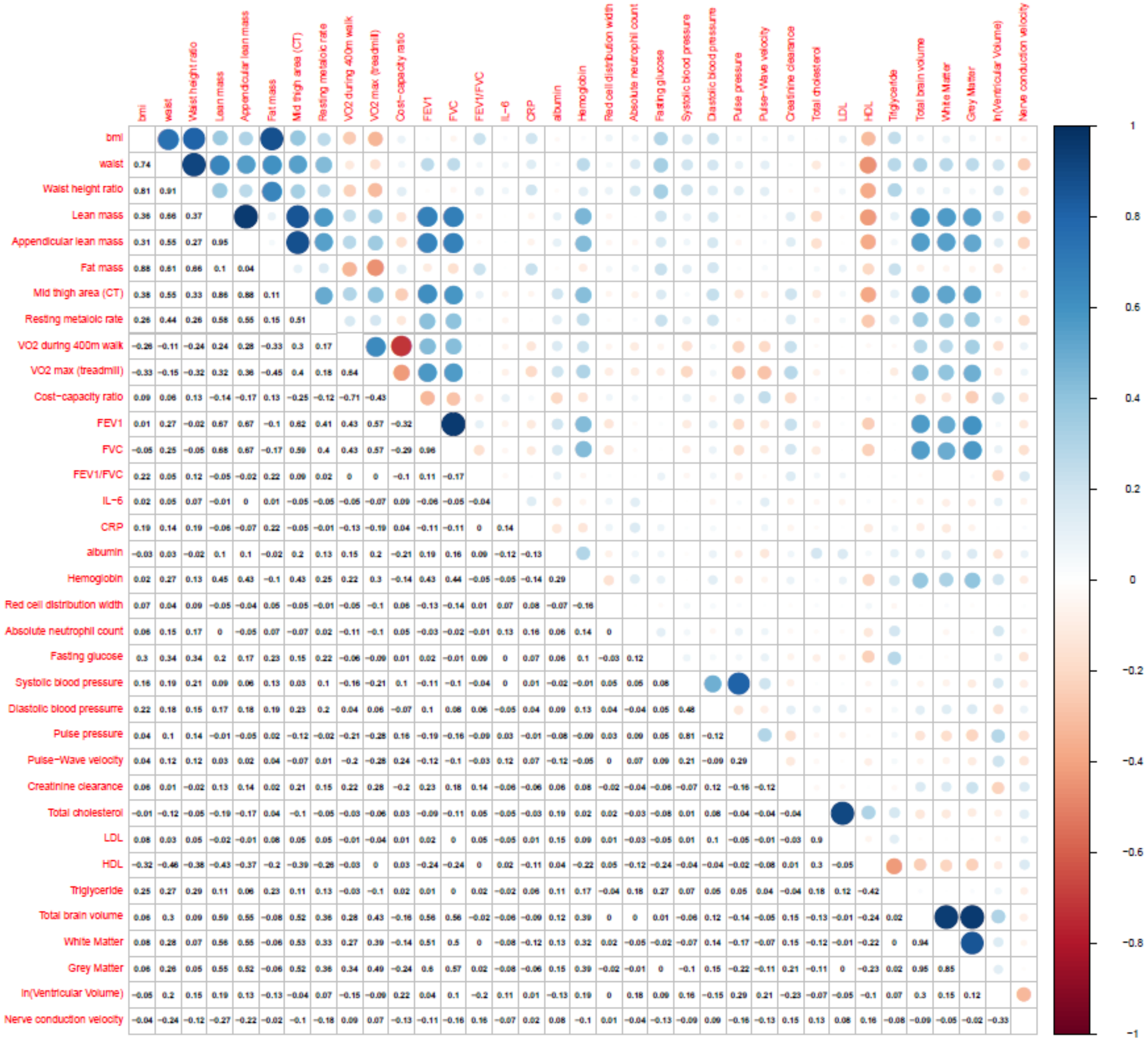

Supplemental Figure 12. Pairwise Cross-sectional Correlation between functional outcomes

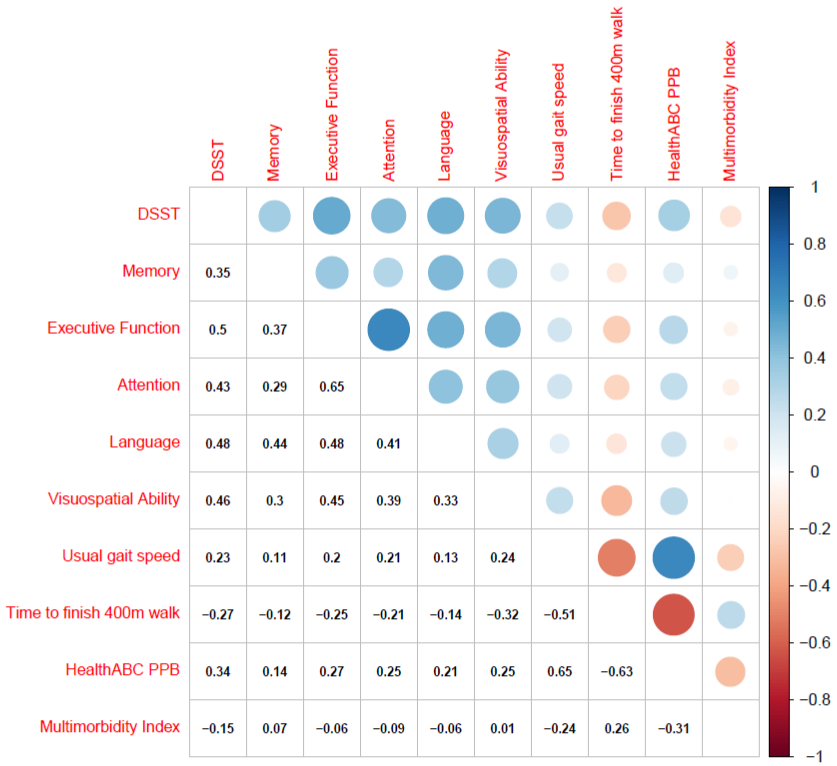

Abbreviation: HealthABC PPB = Health Aging, and Body Composition short Physical Performance Battery

```
############################################################
##### Complementary Codes
##### Creating the global longitudinal phenotypic score
############################################################

### Pre-requisit
## Save the standardized slope difference for each phenotype
## Tips: random slope can be extracted by `ranef` after
## fitting the linear mixed model (with random intercept and slope)
## using `lmer` (under package `lme4`)
## standardization is conducted by
## (1) minus the mean, and then (2) divided by standard deviation



rm(list = ls())
library(dplyr)
library(tidyverse)

## Read in all the files
# dir.out <- "D:/BLSA/data/slopeout"
# each file is for one phenotype
# with the information of `idno` and slope(`stdqqrslope_PHENONAME`)
x <- list.files(dir.out)

### Read in all the slope data data
for(i in 1:length(x)){
  if(i==1){
    merge.all = read.csv(paste0(dir.out,x[i]))
    merge.all$idno = as.integer(merge.all$idno)
```

```
  }else{
    temp = read.csv(paste0(dir.out,x[i]))
    temp$idno = as.integer(temp$idno)
    merge.all = merge.all %>%
      dplyr::full_join(.,temp,by = c("idno"))
    merge.all$idno = as.integer(merge.all$idno)
  }

}

# varlist
bc.list = c("bmi","waist","waist_htcm",
            "leanmass","alm","fatmass","thigh")

ener.list = c("rmr_kcal","vo2kg_400","vo2_max",
              "cost_ratio",
              "fev1","fvc","fev1fvc")

homo.list = c("il6raw","crpraw",
              "albumin","hb","rdw","anc",
              "glucose000_adjusted",
              "sbp","dbp","pp","cfpwv",
              "crcl_cor_use",
              "tc","ldl","hdl","tg")

neuro.list = c("tbcln","wmcln","gmcln","logvv",
               "ncvpf")
```

```
# Example of a selected list within a domain
# Take neuroplasticity/neurodegeneration domain for example
need.list.ex = c("idno",
            paste0("stdqqrslope_",neuro.list))

merge.need.ex = merge.all[names(merge.all) %in% need.list.ex]



# Creating a score
# Take neuroplasticity/neurodegeneration domain for example
merge.use.as.raw = merge.all %>%
  dplyr::select(idno,starts_with("stdqqrslope"))

# Assigning the score depending on the distance from population average
# for each phenotype
merge.use.as.v2 = merge.use.as.raw

for(i in 2:ncol(merge.use.as.v2)){
  # starting from 2 b/c first column is `idno`
  merge.temp <- merge.use.as.raw[,i]
  merge.temp[!is.na(merge.temp) & merge.temp <= -2.5 & merge.temp >= -5] <- -3
  merge.temp[!is.na(merge.temp) & merge.temp <= -1.5 & merge.temp > -2.5] <- -2
  merge.temp[!is.na(merge.temp) & merge.temp <= -0.5 & merge.temp > -1.5] <- -1
  merge.temp[!is.na(merge.temp) & merge.temp < 0.5 & merge.temp > -0.5] <- 0
  merge.temp[!is.na(merge.temp) & merge.temp < 1.5 & merge.temp >=  0.5] <- 1
  merge.temp[!is.na(merge.temp) & merge.temp < 2.5 & merge.temp >=  1.5] <- 2
  merge.temp[!is.na(merge.temp) & merge.temp <= 5 & merge.temp >=  2.5] <- 3
  # In case you need to see how the process goes:
  # table(merge.temp, useNA = "always") %>% print(.)
```

```
  merge.use.as.v2[,i] <- merge.temp
}

# If you want a positive value means accelerated aging
# then you want to compare the population trends and reverse if necessary
# For example, albumin declines over time,
# so if one experienced faster decline,
# you need to flip the sign.
# Here's an example how to flip the sign
merge.use.as.v2$stdqqrslope_albumin = -merge.use.as.v2$stdqqrslope_albmin



# After appropriate flip the sign
# You can create domain-specific and global scores

merge.use.as.v2$score.temp <- NA
merge.use.as.v2$score.b <- NA
merge.use.as.v2$score.n <- NA
merge.use.as.v2$score.e <- NA
merge.use.as.v2$score.h <- NA

# Now we'll use the var list that we listed in the beginning
# bc.list;neuro.list;ener.list;homo.list

for(i in 1:nrow(merge.use.as.v2)){

  # Look at each participant
  score.i <- merge.use.as.v2[i,]
```

```
# select domain-specific phenotpyes
score.i.b <- score.i[names(score.i) %in% paste0("stdqqrslope_",bc.list)]
score.i.n <- score.i[names(score.i) %in% paste0("stdqqrslope_",neuro.list)]
score.i.e <- score.i[names(score.i) %in% paste0("stdqqrslope_",ener.list)]
score.i.h <- score.i[names(score.i) %in% paste0("stdqqrslope_",homo.list)]

temp.x.b <- t(as.vector(score.i.b))
temp.x.n <- t(as.vector(score.i.n))
temp.x.e <- t(as.vector(score.i.e))
temp.x.h <- t(as.vector(score.i.h))

# Use all available phenotypes to create the domain-specific scores
mean.score.i.b <- mean(temp.x.b, na.rm = TRUE)
mean.score.i.n <- mean(temp.x.n, na.rm = TRUE)
mean.score.i.e <- mean(temp.x.e, na.rm = TRUE)
mean.score.i.h <- mean(temp.x.h, na.rm = TRUE)

# Note:
# missing domain-available score is allowed in this temp step
# We will restrict to those with all four domain-available later on
mean.score.i.a <- mean(c(mean.score.i.b,mean.score.i.n,
                         mean.score.i.e,mean.score.i.h),
                       na.rm = TRUE)

merge.use.as.v2$score.b[i] <- mean.score.i.b
merge.use.as.v2$score.n[i] <- mean.score.i.n
merge.use.as.v2$score.e[i] <- mean.score.i.e
merge.use.as.v2$score.h[i] <- mean.score.i.h
merge.use.as.v2$score.temp[i] <- mean.score.i.a ## across domains
```

```
}

temp <- merge.use.as.v2 %>%
  dplyr::rename(acchigh_score.temp = score.temp,
                # higher value is accelerated aging
                acchigh_score.b = score.b,
                acchigh_score.e = score.e,
                acchigh_score.h = score.h,
                acchigh_score.n = score.n)

# Do quantile normalization for each domain
# This is important because number of phenotpyes vary across domain!
# This step can put them on the same scale

temp$acchigh_qq.b <- NA;
temp$acchigh_qq.b[!is.na(temp$acchigh_score.b)] <-
qqnorm(temp$acchigh_score.b[!is.na(temp$acchigh_score.b)])$x

temp$acchigh_qq.e <- NA;
temp$acchigh_qq.e[!is.na(temp$acchigh_score.e)] <-
qqnorm(temp$acchigh_score.e[!is.na(temp$acchigh_score.e)])$x

temp$acchigh_qq.h <- NA;
temp$acchigh_qq.h[!is.na(temp$acchigh_score.h)] <-
qqnorm(temp$acchigh_score.h[!is.na(temp$acchigh_score.h)])$x

temp$acchigh_qq.n <- NA;
temp$acchigh_qq.n[!is.na(temp$acchigh_score.n)] <-
qqnorm(temp$acchigh_score.n[!is.na(temp$acchigh_score.n)])$x
```

```
# `temp.out` and `simple.out` are what we need
temp.out = temp %>%
  mutate(acchigh_score.qq = rowMeans(select(., starts_with("acchigh_qq.")),
                                     na.rm = FALSE),
         # Note: 'na.rm = FALSE' here
         # This will make those without all 4 domain-specific scores
         # become NA.
         # `acclow_score.qq` is simply flipping the sign
         # so that lower score means accelerated aging
         # People have different feelings about it,
         # and you can make your own decision.
         acclow_score.qq = -acchigh_score.qq)


simple.out = temp.out %>%
  # Now, we keep only those with four domain-specific scores available
  # into our final analytic dataset
  # We integetrate the scheme of four phenotypic domains and
  # all the available longitudinal trajectories.
  # Note that we do pay some price here.
  # We give all the phenotypes equal weights within domain,
  # and we give all four domains equal weights for the construction
  # of final score. We also have some Bayesian mindset with
  # the prior that some unmeasured phenotypes can be presented by
  # other measured phenotypes for the construction
  # of domain-specific score.
  dplyr::filter(!is.na(acchigh_score.qq)) %>%
  dplyr::select(idno,starts_with("acc"))
```

```
# You can save the scores and do more aging research.

write.csv(simple.out, PATH)
```
