# Supplementary material for: Longitudinal phenotypic aging metrics in the Baltimore Longitudinal Study of Aging
Source: Nat Aging. 2022 Jul 18;2(7):635–43. doi: 10.1038/s43587-022-00243-7 (PMC9997119; doi:10.1038/s43587-022-00243-7)
Supplement: Supplementary file 2 — Reporting Summary [file 43587_2022_243_MOESM2_ESM.pdf]

nature portfolio

Corresponding author(s): DBPR, NATAGING-A01337A-Z
Luigi Ferrucci, MD, PhD
National Institute on Aging, NIH
Baltimore, MD

Last updated by author(s): May 5, 2022

Reporting Summary

Nature Portfolio wishes to improve the reproducibility of the work that we publish. This form provides structure for consistency and transparency in reporting. For further information on Nature Portfolio policies, see our Editorial Policies and the Editorial Policy Checklist.

Statistics

For all statistical analyses, confirm that the following items are present in the figure legend, table legend, main text, or Methods section.

n/a	Confirmed	
☐	☒	The exact sample size (*n*) for each experimental group/condition, given as a discrete number and unit of measurement
☐	☒	A statement on whether measurements were taken from distinct samples or whether the same sample was measured repeatedly
☐	☒	The statistical test(s) used AND whether they are one- or two-sided<br>*Only common tests should be described solely by name; describe more complex techniques in the Methods section.*
☐	☒	A description of all covariates tested
☐	☒	A description of any assumptions or corrections, such as tests of normality and adjustment for multiple comparisons
☐	☒	A full description of the statistical parameters including central tendency (e.g. means) or other basic estimates (e.g. regression coefficient) AND variation (e.g. standard deviation) or associated estimates of uncertainty (e.g. confidence intervals)
☐	☒	For null hypothesis testing, the test statistic (e.g. *F*, *t*, *r*) with confidence intervals, effect sizes, degrees of freedom and *P* value noted<br>*Give P values as exact values whenever suitable.*
☒	☐	For Bayesian analysis, information on the choice of priors and Markov chain Monte Carlo settings
☐	☒	For hierarchical and complex designs, identification of the appropriate level for tests and full reporting of outcomes
☒	☐	Estimates of effect sizes (e.g. Cohen's *d*, Pearson's *r*), indicating how they were calculated

Our web collection on statistics for biologists contains articles on many of the points above.

Software and code

Policy information about availability of computer code

Data collection	Data was collected via the platform OpenClinica (https://www.openclinica.com/)
Data analysis	All the analysis was performed using R 3.6.2, Stata version 15.1 (StataCorp, College Station, Texas), and SAS 9.4. The R packages used for data wrangling and analysis include \`dplyr\`(v0.8.5), \`tidyverse\` (v1.3.0), \`survival\` (v3.2.7), \`flexsurv\` (v1.1.1), \`lme4\`(v1.1.23). Epigenetic ages were processed with \`minfi\`(Aryee et al. 2014) calculated using the Horvath online calculator (https://dnamage.genetics.ucla.edu/) or “projector” package (https://github.com/danbelsky/DunedinPoAm38).

For manuscripts utilizing custom algorithms or software that are central to the research but not yet described in published literature, software must be made available to editors and reviewers. We strongly encourage code deposition in a community repository (e.g. GitHub). See the Nature Portfolio guidelines for submitting code & software for further information.

Data

Policy information about availability of data

All manuscripts must include a data availability statement. This statement should provide the following information, where applicable:

- Accession codes, unique identifiers, or web links for publicly available datasets
- A description of any restrictions on data availability
- For clinical datasets or third party data, please ensure that the statement adheres to our policy

Data are available upon request through the BLSA website (https://www.blsa.nih.gov/).

Field-specific reporting

Please select the one below that is the best fit for your research. If you are not sure, read the appropriate sections before making your selection.

☒ Life sciences ☐ Behavioural & social sciences ☐ Ecological, evolutionary & environmental sciences

For a reference copy of the document with all sections, see nature.com/documents/nr-reporting-summary-flat.pdf

Life sciences study design

All studies must disclose on these points even when the disclosure is negative.

Sample size	Sample size is not predetermined. We used all the data collected in BLSA for this analysis.
Data exclusions	Because the aim is to calculate the global longitudinal phenotypic score across four domains, BLSA participants included needed to have longitudinal trajectories across four pre-hypothesized domains (at least one phenotype measured longitudinally for each domain). Details have are reported in the manuscript.
Replication	BLSA is unique because of its longitudinal comprehensive measurements based on a pre-hypothesized classification of the metrics of aging. Thus, we did not replicate this analysis in an independent cohort.
Randomization	This is an observational cohort study, so the potential confounders are adjusted in our regression model.
Blinding	This is an observational cohort study, so blinding is not relevant.

Reporting for specific materials, systems and methods

We require information from authors about some types of materials, experimental systems and methods used in many studies. Here, indicate whether each material, system or method listed is relevant to your study. If you are not sure if a list item applies to your research, read the appropriate section before selecting a response.

Materials & experimental systems

n/a	Involved in the study	
☒	☐	Antibodies
☒	☐	Eukaryotic cell lines
☒	☐	Palaeontology and archaeology
☒	☐	Animals and other organisms
☐	☒	Human research participants
☐	☒	Clinical data
☒	☐	Dual use research of concern

Methods

n/a	Involved in the study	
☒	☐	ChIP-seq
☒	☐	Flow cytometry
☒	☐	MRI-based neuroimaging

Human research participants

Policy information about studies involving human research participants

Population characteristics	Participants with longitudinal phenotypic measurements across four pre-identified domains were included (n = 968). Among these 968 participants, 512 (52.9%) were women, and baseline age ranged between 24.9 and 93.7 years, with a median follow-up around 7-9 years.
Recruitment	BLSA is an ongoing longitudinal study of aging. Participants who are free of major diseases and are enrolled and assessed longitudinally. Details about inclusion and exclusion criteria used in the recruitment can be found on BLSA website (https://www.blsa.nih.gov/) and our previous publication (https://www.ncbi.nlm.nih.gov/pmc/articles/PMC7670826/).
Ethics oversight	Internal Review Board of National Institutes of Health, Bethesda, USA

Note that full information on the approval of the study protocol must also be provided in the manuscript.

Clinical data

Policy information about clinical studies

All manuscripts should comply with the ICMJE guidelines for publication of clinical research and a completed CONSORT checklist must be included with all submissions.

Clinical trial registration	https://clinicaltrials.gov/ct2/show/NCT00233272
Study protocol	Full study protocol can be found on BLSA website (https://www.blsa.nih.gov/). In brief, the BLSA is a study of normative human aging,

Study protocol

established in 1958, comprehensively revised in 2003 with more extensive domain-based phenotypic measurements, and conducted by the National Institute on Aging Intramural Research Program. All participants are community-dwelling volunteers free of major chronic conditions upon enrollment. Detailed inclusion/exclusion criteria are described in our previous work (https://www.ncbi.nlm.nih.gov/pmc/articles/PMC7670826/). Enrolled participants are asked to stay in our clinical site (current clinical site: NIA site at Harbor Hospital) for three days for assessments performed by trained and certified technicians following standardized protocols with the provided written informed consent at each visit.

Data collection

Enrolled participants are followed up with an age-dependent frequency (<60 every 4 years, 60-79 every 2 years, > 80 every year) to account for the faster functional decline at the later part of life. All assessments were collected by trained and certified technicians following standardized protocol. The analytic sample for the current study mainly consists of participants who underwent repeated phenotypic measurements during their clinic visits between January 2005 and December 2019.

Outcomes

The outcomes are longitudinal changes in physical and cognitive functions, accumulation of multi-morbidity, and mortality. Physical function was evaluated through objective performance measures of mobility; cognitive function was evaluated through validated cognitive testing administered by expert interviewers. Multi-morbidity was evaluated as the number of medical conditions reported by participants. Mortality was ascertained by regular contact with participants and consultation of the National Death Index. Details on these measures are reported in details as a supplement to the manuscript.
